# Untargeted Metabolomic Characterization of Ovarian Tumors

**DOI:** 10.3390/cancers12123642

**Published:** 2020-12-04

**Authors:** Xiaona Liu, Gang Liu, Lihua Chen, Fei Liu, Xiaozhe Zhang, Dan Liu, Xinxin Liu, Xi Cheng, Lei Liu

**Affiliations:** 1 Institutes of Biomedical Sciences, Fudan University, Shanghai 200032, China; 16111520003@fudan.edu.cn (X.L.); liugang@fudan.edu.cn (G.L.); 2 Department of Gynecological Oncology, Fudan University Shanghai Cancer Center, Department of Oncology, Shanghai Medical College, Fudan University, Shanghai 200032, China; 19111230043@fudan.edu.cn (L.C.); 13211230024@fudan.edu.cn (F.L.); 3 CAS Key Laboratory of Separation of Science for Analytical Chemistry, Dalian Institute of Chemical Physics, Chinese Academy of Sciences, Dalian 116023, China; zhangxz@dicp.ac.cn (X.Z.); liudan@dicp.ac.cn (D.L.); liuxinxin@dicp.ac.cn (X.L.); 4 Data Science, School of (Institute for Big Data), Fudan University, Shanghai 200032, China; 5 Academy for Engineering and Technology, Fudan University, Shanghai 200032, China; 6 Faculty of Medical Instrumentation, Shanghai University of Medicine and Health Sciences, Shanghai 201318, China

**Keywords:** ovarian tumors, metabolomics, diagnosis, early diagnosis, urine, plasma

## Abstract

**Simple Summary:**

This study utilized untargeted metabolomic techniques to detect urine and plasma metabolites. Using support vector machine algorithm, three models for ovarian tumors diagnosis, benign-malignant distinguishing, early diagnosis and borderline-malignant distinguishing were developed. These models have good classification performance and provided a novel insight for non-invasive diagnosis of ovarian cancer.

**Abstract:**

Diagnosis of ovarian cancer is difficult due to the lack of clinical symptoms and effective screening algorithms. In this study, we aim to develop models for ovarian cancer diagnosis by detecting metabolites in urine and plasma samples. Ultra-high-performance liquid chromatography and quadrupole time-of-flight mass spectrometry (UHPLC-QTOF-MS) in positive ion mode was used for metabolome quantification in 235 urine samples and 331 plasma samples. Then, Urine and plasma metabolomic profiles were analyzed by univariate and multivariate statistics. Four groups of samples: normal control, benign, borderline and malignant ovarian tumors were enrolled in this study. A total of 1330 features and 1302 features were detected from urine and plasma samples respectively. Based on two urine putative metabolites, five plasma putative metabolites and five urine putative metabolites, three models for distinguishing normal-ovarian tumors, benign-malignant (borderline + malignant) and borderline-malignant ovarian tumors were developed respectively. The AUC (Area Under Curve) values were 0.987, 0876 and 0.943 in discovery set and 0.984, 0.896 and 0.836 in validation set for three models. Specially, the diagnostic model based on 5 plasma putative metabolites had better early-stage diagnosis performance than CA125 alone. The AUC values of the model were 0.847 and 0.988 in discovery and validation set respectively. Our results showed that normal and ovarian tumors have unique metabolic signature in urine and plasma samples, which shed light on the ovarian cancer diagnosis and classification.

## 1. Introduction

Ovarian cancer (OC) is one of the most lethal cancer among gynecological malignancies with 294,414 new ovarian cancer cases and 184,799 ovarian cancer deaths worldwide in 2018 [1]. The International Agency for Research on Cancer (IARC) expected 73,300 new cases of ovarian cancer in China in 2018, ranking 10th in Chinese women’s malignancies [2]. Because of lacking early symptoms, 80% of women with OC are in advanced stages at the time of initial diagnosis [3]. The survival rate of ovarian cancer is highly related to tumor stage. The 5-year survival rate of FIGO (International Federation of Gynecology and Obstetrics) I stage patients is as high as 92%, while that of FIGO IV stage patients is only 5% [3]. Thus, early diagnosis is crucial to improve the prognosis of ovarian carcinoma. Currently, the most commonly used clinical biomarkers for ovarian cancer are CA125 (Cancer Antigen 125) and HE4 (Human Epididymis Protein 4). However, the specificity and sensitivity are relatively unsatisfactory [4], which makes it necessary to research novel biomarkers or model for the diagnosis even early diagnosis of ovarian cancer.

As one of the hallmarks of tumor, metabolic alteration has been emphasized in the past decades [5]. Metabolomics is an overall assessment of endogenous small molecule metabolites in biological systems, with the potential to identify important metabolic changes in various diseases [6]. It has been used to find new diagnostic markers and explore pathogenesis in ovarian cancer and other cancers [7,8,9,10]. Previous studies had suggested that exploring the metabolic characteristics of biological samples could facilitate clinical diagnosis of ovarian cancer and understand the underlying biological mechanisms of ovarian cancer. However, the sample size of previous ovarian cancer metabolomics researches were relatively small and some lacked external validation sets [11,12], which limited the robustness and statistical significance of the results. In addition, some studies discriminating malignant and normal samples ignored the bias brought by benign samples [7,11], which may increase false positive rates. There were overmuch ions in the diagnostic model in some research, which increases the complexity of the model and the probability of over-fitting [11,13,14]. The research about the different metabolite patterns between ovarian carcinomas and ovarian borderline tumors is very few [15].

In this work, ultra-high-performance liquid chromatography and quadrupole time-of-flight mass spectrometry (UHPLC-QTOF-MS) was used to identify the altered metabolites between normal controls, benign, borderline and malignant ovarian tumors in urine and plasma samples. The significantly dysregulated metabolic patterns were detected and models for ovarian cancer diagnosis were developed. Compared with clinically used biomarkers and other reported panels, the models in this study have satisfactory performance in identifying different types of ovarian tumors by detecting metabolites in plasma or urine samples.

## 2. Results

### 2.1. Sample Enrollment

There were 76 normal, 50 benign, 14 borderline and 95 malignant plasma samples and 80 normal, 81 benign, 20 borderline and 150 malignant urine samples which were prospectively collected and enrolled in this study. In some subsequent analyses, borderline tumors were classified into the malignant tumor group, which will be labeled. In the borderline and malignant plasma samples, the sample numbers of FIGO (International Federation of Gynecology and Obstetrics) I, II, III and IV were 18, 14, 67 and 7, respectively, with median age of 56 years old. In the borderline and malignant urine samples, the numbers were 35, 17, 104 and 12, respectively, with median age of 54 years old. To narrow down the genetic and environmental disturbance of individuals, paired urine and plasma samples were collected from 148 patients. The detailed clinical characteristics of urine and plasma samples enrolled in this study were shown in Table 1 and Table S1. No significant difference existed between urine and plasma samples on clinical indicators. The samples in this study were divided into discovery and validation cohorts. The urine/plasma paired samples were used for discovery set and the other unpaired samples were used for validation set. The discovery cohort includes 40 health, 36 benign, 13 borderline and 74 malignant samples. The technique pipeline of this study was shown in Figure S1.

### 2.2. Metabolic Profile of Urine and Plasma in Ovarian Carcinoma

After data processing, including metabolite signature screening, missing value filling and median normalization, 1330 and 1302 metabolic features were detected from urine and plasma samples respectively. Principle component analysis (PCA) was used to visualize the data quality and metabolic profile of plasma and urine samples. As shown in Figures S2 and S3, all quality control (QC) samples were tightly clustered together compared to other samples. In addition, the top two components could effectively discriminate the normal and ovarian tumor urine samples, in regardless of cohorts and upper/lower phase (Figure S2). However, the performance of two components in plasma was not as favorable as the urine samples (Figure S3), which indicated that the difference metabolic profile between urine and plasma samples. In another method, we added exogenous quercetin (RT-m/z, PL: 1.8607-303.0509, PU: 6.6644-303.0501, UL: 1.8136-303.0471) as an internal standard in the analysis for assessing data reliability. The relative standard deviations (RSDs) of quercetin in QC samples were 54.06 and 42.04 in plasma nonpolar sample (PL), 56.11 and 23.51 in plasma polar sample (PU), 80.99 and 10.69 in urine nonpolar sample (UL) of the first and second batches of samples respectively. For urine polar sample (UU) results, we selected five representative ion peaks (covering different retention times and different intensities) from the total ion current map of QC samples (RT-m/z: 0.4902-212.8509, 5.0416-505.2678, 6.0261-225.133, 7.8678-175.0757, 9.7199-287.1794). The RSDs of these ion peaks in QC samples were 24.11, 22.15, 30.1, 27.2 and 32.4 in the first batch of samples, 12.6, 39.45, 38.73, 7.54 and 12.95 in the second batch of samples. These results indicated that the UHPLC-QTOF analytical system had good stability and the data quality was creditable for subsequent assay.

**Figure 1:**
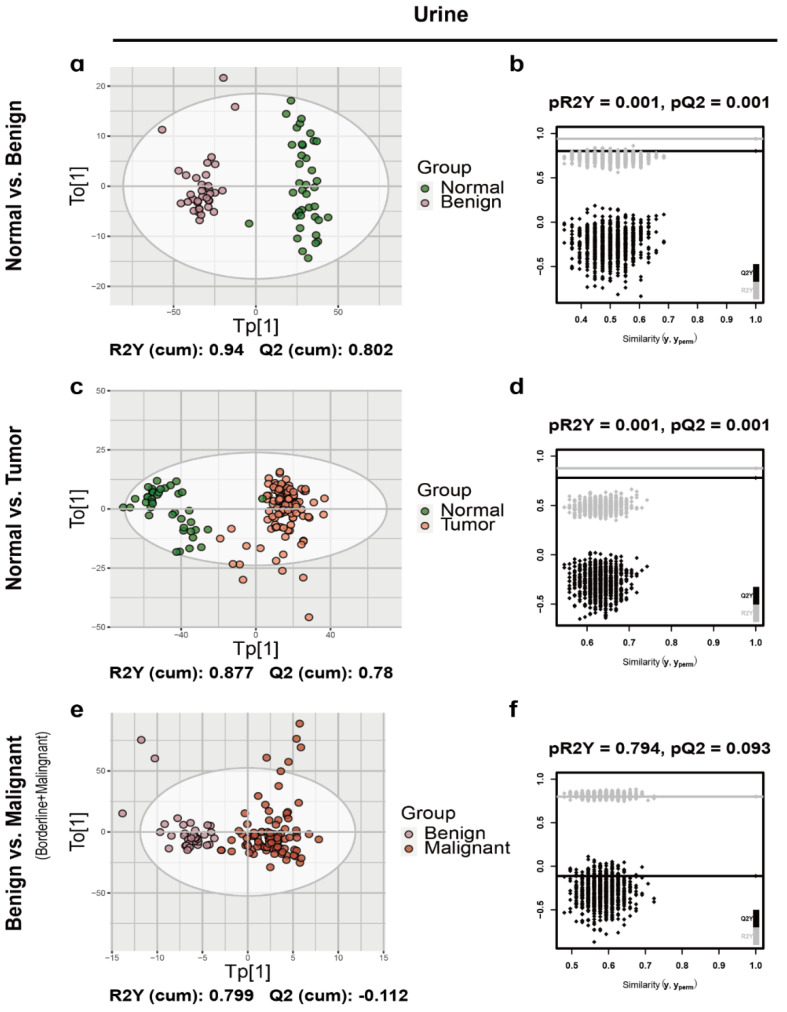
The orthogonal partial least-squared discriminant analysis (OPLS-DA) score plot and the permutation test results of urine samples. The OPLS-DA score plot between (**a**) normal and benign, (**c**) normal and ovarian tumor and (**e**) benign and malignant (borderline + malignant) ovarian tumors in plasma samples and the corresponding permutation test results (**b**,**d**,**f**).

**Figure 2:**
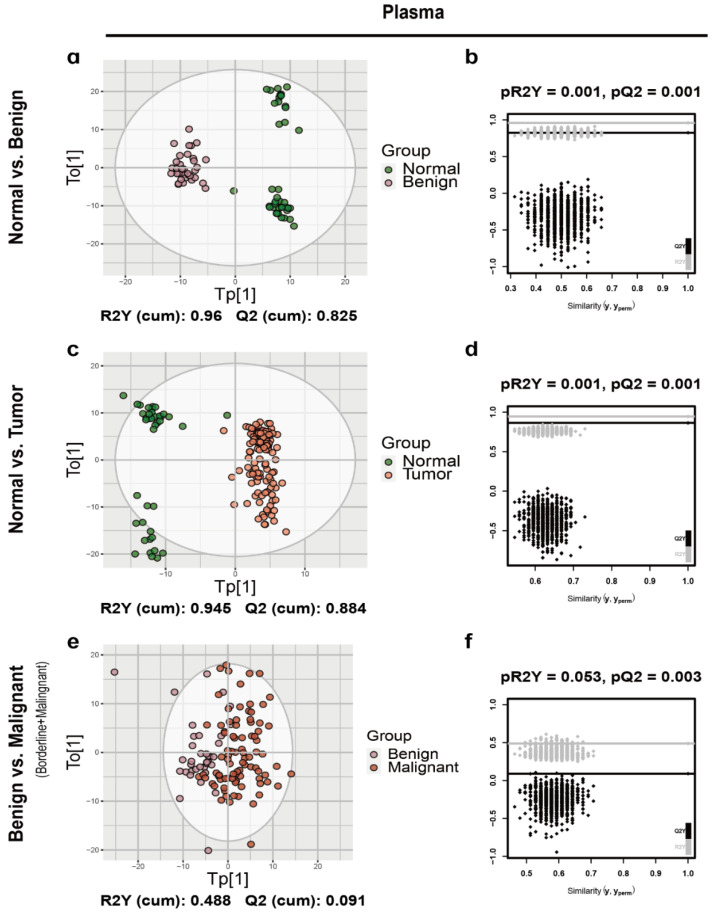
The OPLS-DA score plot and the permutation test results of plasma samples. The OPLS-DA score plot between (**a**) normal and benign, (**c**) normal and ovarian tumor and (**e**) benign and malignant (borderline + malignant) ovarian tumors in urine samples and the corresponding permutation test results (**b**,**d**,**f**).

After splitting the data to discovery and validation sets, the orthogonal partial least-squared discriminant analysis (OPLS-DA) was implemented to characterize metabolic patterns in the urine and plasma discovery cohort (Figure 1 and Figure 2, Figures S4 and S5). As expected, urine normal samples were separated from benign (Figure 1a), borderline (Figure S4a) and malignant tumor samples (Figure S4c) with R2Y and Q2 greater than 0.5, which was consistent with the results of plasma (Figure 2a and Figure S5a,c). Additionally, normal samples were separated well from ovarian tumor samples with R2Y and Q2 greater than 0.5 in both urine and plasma (Figure 1c and Figure 2c). So, we first focused on the comparation between normal controls and ovarian tumors. As shown in Figure 1, the urine OPLS-DA model between benign and malignant (borderline + malignant) was overfitting (pQ2 = 0.093) (Figure 1f), while plasma OPLS-DA model between benign and malignant (borderline + malignant) was not overfitting (pQ2 = 0.003) (Figure 2f). Thus, the OPLS-DA model based on plasma metabolic features allowed to distinguish ovarian tumor classes. There were sufficient borderline ovarian tumor urine samples in discovery and validation cohorts. Metabolic difference between benign and malignant (borderline + malignant) ovarian tumors, borderline and malignant ovarian tumors (Figures S4e and S5e) were also compared.

### 2.3. Differential Putative Metabolites and Category

**Figure 3:**
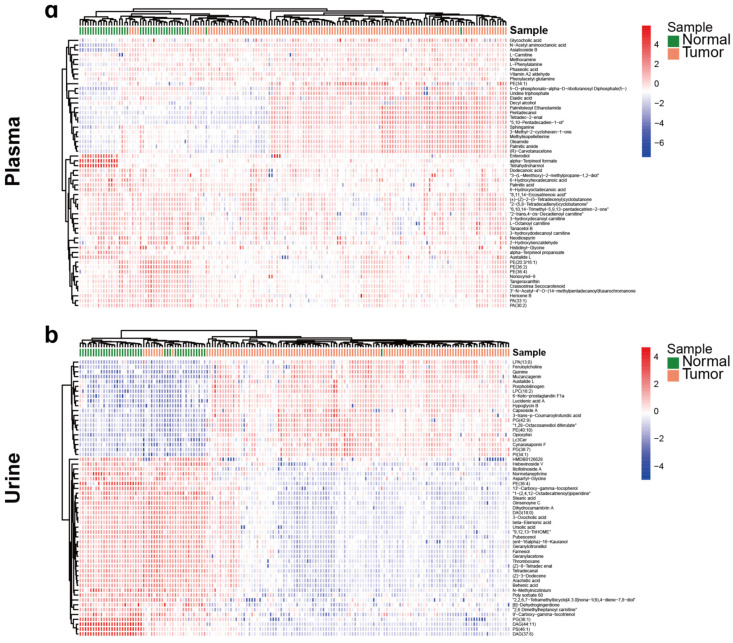
Heatmap of the differential putative metabolites from normal controls versus ovarian tumors. (**a**) Heatmap of the differential putative metabolites from normal control versus ovarian tumor in urine samples. (**b**) Heatmap of the differential putative metabolites from normal control versus ovarian tumor in plasma samples. Red color indicated higher level in ovarian tumor. Blue color indicated lower level in ovarian tumor.

The differential urine putative metabolites between normal and ovarian tumor samples were identified and 504 metabolic features were significantly different in urine, among which, 57 differential features could be identified as putative metabolites (Table S2). The relative quantities of these differential putative metabolites between normal and ovarian tumor samples were shown in a heatmap (Figure 3a). Similar pattern was detected in plasma samples (Figure 3b), with 223 differential metabolic features and 56 detectable differential putative metabolites (Table S3). These putative metabolites discriminated the normal and ovarian tumor samples well. A total of 152 and 101 differential metabolic features between benign and malignant (borderline and malignant) ovarian tumors also were detected in urine and plasma respectively and 53 and 19 differential putative metabolites were identified respectively (Tables S2 and S3). There were 64 and 112 differential metabolic features between borderline and malignant ovarian tumors in urine and plasma respectively, among which, 12 and 10 differential putative metabolites were identified respectively (Tables S2 and S3).

Differential putative metabolites were categorized by searching the Human Metabolome Database (HMDB, http://www.hmdb.ca) [16]. The results showed that the differential urine putative metabolites of three comparison groups (normal vs. ovarian tumor, benign vs. malignant (borderline + malignant), borderline vs. malignant) were mainly lipids and lipid-like molecules (73.68%, 62.75%, 50%, respectively) and the second largest class of putative metabolites were organic acids and derivatives in the normal versus ovarian tumor and benign versus malignant (borderline + malignant) comparison group (7.02%, 9.8%, respectively) (Figure S6a,b), while organoheterocyclic compounds was the second largest class of putative metabolites in borderline versus malignant comparison group (25%) (Figure S6c). Same as urine putative metabolites, plasma differential putative metabolites were concentrated in class of lipids and lipid-like molecules (62.5% in normal versus ovarian tumor, 52.63% in benign versus malignant, 40% in borderline versus malignant) and the second largest class was organic acids and derivatives in normal versus ovarian tumor and borderline versus malignant comparison groups (7.14%, 10%, respectively) (Figure S6d,f), while organic oxygen compounds was the second largest class of putative metabolites in benign versus malignant (borderline + malignant) comparison group (15.79%) (Figure S6e).

### 2.4. Diagnostic Model

**Figure 4:**
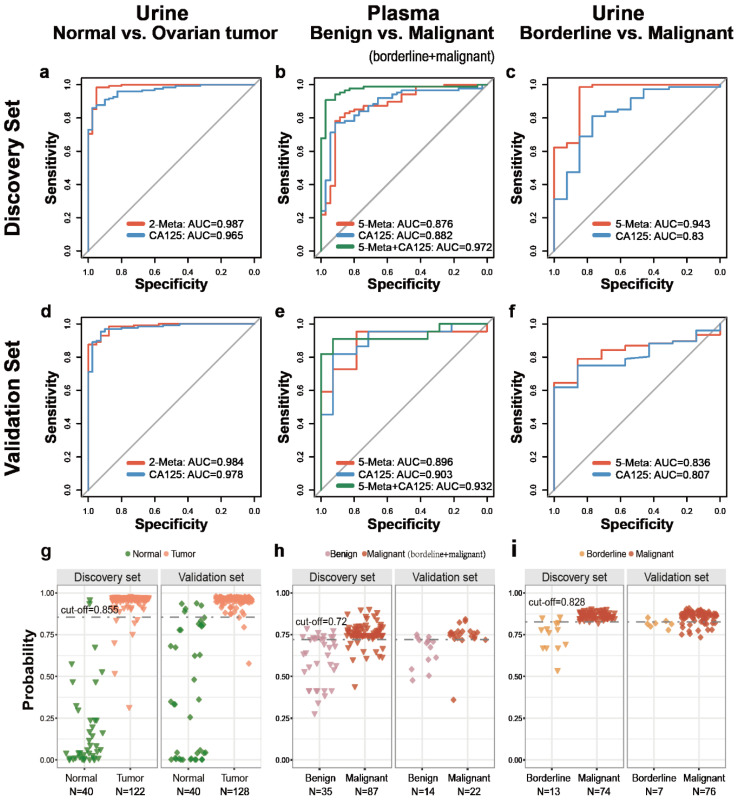
Diagnostic performance and prediction plot of biomarkers in urine or plasma sample. The diagnostic performance in the discovery set and validation set were shown via the receiver-operating characteristic (ROC) curves for comparison between (**a**,**d**) normal control versus ovarian tumors, (**b**,**e**) benign versus malignant (borderline + malignant), (**c**,**f**) borderline versus malignant ovarian tumors. The prediction accuracies by the biomarkers in the discovery set and validation set were compared between (**g**) normal control versus ovarian tumor, (**h**) benign versus malignant (borderline + malignant), (**i**) borderline versus malignant ovarian tumor. AUC = area under the curve. Red line indicates biomarker panel. blue line indicates CA125. Green line indicates the hybrid model based on biomarker panel and CA125.

The optimal support vector machine (SVM) classification models based on the best biomarker combination were constructed as described in Method section. As summarized in Table 2, there were 2 urine putative metabolic biomarkers for distinguishing normal from ovarian tumors, 5 plasma putative metabolic biomarkers for benign versus malignant, 5 urine putative metabolic biomarkers for borderline versus malignant. The receiver operating characteristic (ROC) curves, based on the SVM model of each biomarker panel from the discovery set were shown in Figure 4 and Table 3; AUC (the areas under the curve), sensitivity and specificity were 0.987 (Figure 4a), 94.26%, 95.0% (Figure 4g and Table 3, higher or similar than 0.965, 87.70% and 95% of CA125 alone) for normal versus ovarian tumor (N = 162; seed = 1000, gamma = 0.1, cost = 100); 0.876 (Figure 4b), 87.36%, 62.86% (Figure 4h and Table 3) for benign versus malignant (borderline + malignant) (N = 122; seed = 520, gamma = 0.1, cost = 10); 0.943 (Figure 4c), 98.65%, 84.62% (Figure 4i and Table 3) ((higher than 0.830, 81.08% and 76.92% of CA125 alone)) for borderline versus malignant (N = 87; seed = 520, gamma = 0.1, cost = 10), respectively. Based on the highest prediction sensitivity and specificity of the ROC in the discovery phase, the optimal cut-off values were 0.855 for normal versus ovarian tumors, 0.72 for benign versus malignant (borderline + malignant), 0.828 for borderline versus malignant (Table 3). The cut-off values were used to predict the different status of ovarian tumors in the validation set. the AUC, sensitivity and the specificity were 0.984 (higher than 0.978 of CA125 alone) (Figure 4d), 97.66% (higher than 89.84% of CA125 alone), 87.50% (Figure 4g and Table 3) for normal versus ovarian tumor (N = 168); 0.896 (Figure 4e), 86.36%, 78.57% (Figure 4h and Table 3) for benign versus malignant (borderline + malignant) (N = 36); 0.836 (higher than 0.807 of CA125 alone) (Figure 4f), 80.26%, 71.43% (higher than 42.86% of CA125 alone) (Figure 4i and Table 3) for borderline versus malignant (N = 83), respectively.

### 2.5. Diagnosis Performance of Biomarkers Combined to Clinical Indicator

CA125 is a widely used diagnostic marker in clinical. We also assessed the classification accuracy of the hybrid SVM model based both CA125 and putative metabolites biomarkers, relative to CA125 alone. The hybrid SVM model based on CA125 and five plasma putative metabolites for distinguishing benign and malignant (borderline + malignant) ovarian tumors showed a higher diagnostic performance than that of CA125 alone (AUC: 0.972 versus 0.882, 0.932 versus 0.903, in discovery and validation cohorts respectively) (Figure 4b,e). With the cut-off of 0.71, this model had higher sensitivity (93.10% and 90.91% in discovery and validation cohorts, respectively) than CA125 alone (88.51% and 86.36% in discovery and validation cohorts, respectively), while higher specificity (91.43% and 92.86% in discovery and validation cohorts, respectively) than CA125 alone (cut-off: 35, 65.71%, 71.43% in discovery and validation cohorts, respectively) (Figure S7a and Table 4). The effect s of SVM models based on other two biomarker panels and CA125 did not improve greatly than biomarker panels or CA125 alone, which were not shown in article. Overall, the SVM model based on CA125 and 5 plasma putative metabolites had a better diagnostic performance.

### 2.6. Early Diagnosis of Ovarian Carcinoma

**Figure 5:**
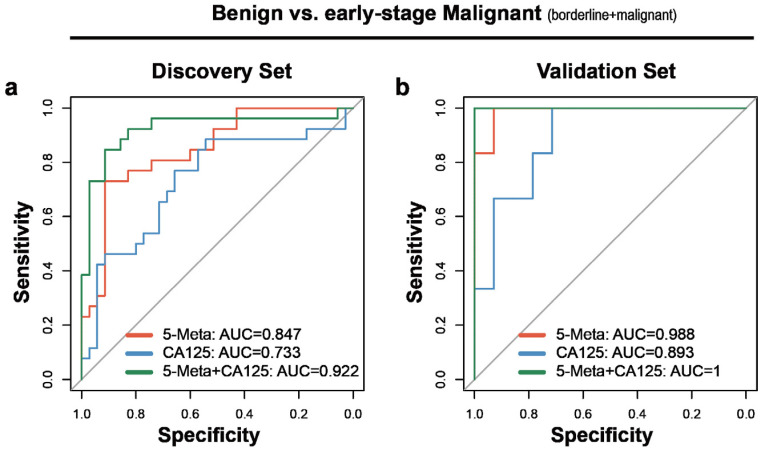
Early-stage diagnostic performance of biomarkers between benign and malignant ovarian tumors in plasma samples. (**a**) The ROC (receiver operating characteristic) curves for comparison between benign versus early-stage ovarian cancer in the discovery set. (**b**) The ROC curves for comparison between benign versus early-stage ovarian cancer in the validation set. AUC = area under the curve. Red line indicates biomarker panel. blue line indicates CA125. Green line indicates the hybrid model based on biomarker panel and CA125.

Early diagnosis of ovarian carcinoma benefits survival. Thus, the performance of this model in FIGO I and II staged samples was assayed. As expected, the AUC of the plasma SVM model based on five putative metabolites in diagnosing the early-stage ovarian carcinoma reached 0.847and 0.988 in discovery and validation sets respectively, which were higher than 0.733 and 0.893 of CA125 alone (Figure 5a,b). This model had improved sensitivity than CA125 alone in discovery and validation sets (80.77% versus 69.23%, 100% versus 83.33%, respectively), while the specificity in validation set was higher than CA125 (78.57% versus 71.43%) (Table 4). The hybrid model based on CA125 and five plasma putative metabolites improved the AUC, sensitivity and specificity than CA125 alone and a panel of five putative metabolites (AUC: 0.922, sensitivity: 80.77%, specificity: 91.43%, in discovery set; AUC: 1, sensitivity: 100% and specificity: 92.86 in validation set, respectively) (Figure 5a,b; Table 4).

These results indicated that this plasma SVM models had better performance for diagnosing early-stage ovarian carcinoma than the clinically used CA125 and the hybrid model significantly improved the diagnostic performance.

## 3. Discussion

This study systematically described the metabolomics changes in plasma and urine between normal controls, benign, borderline and malignant ovarian tumor samples using large size and multiple groups for the first time. Most of the previous studies focused only on the difference between ovarian carcinoma and normal samples, malignant and benign ovarian tumors, malignant and benign plus normal samples and did not pay attention to the difference between benign ovarian tumors and normal controls. We first found that a high coincidence rate between the differential putative metabolites between normal controls and OC and the differential putative metabolites between normal controls and BOT (coincident differential putative metabolites/differential putative metabolites: 45/77 in benign versus normal in urine, 45/57 in malignant versus normal samples in urine; 47/49 in benign versus normal samples in plasma, 47/62 in malignant versus normal samples in plasma) and there were more differential putative metabolites between normal and benign samples than between normal and malignant samples in urine (77 versus 57). However, previous study on metabolomics analysis of 26 ovarian cancer, 25 benign ovarian tumor and 25 age-matched control samples showed no differential metabolites between benign ovarian tumor and normal controls [17]. According to results, normal controls and ovarian tumors were significant different at the metabolites level but the difference between benign and malignant ovarian tumors was not obvious. Borderline ovarian tumors are neoplasms of epithelial origin characterized by atypical proliferation and used to be called tumors of low malignant potential but without destructive stromal invasion [18]. We found that the metabolites levels of borderline ovarian tumors did not increase or decrease according to the benign, borderline and malignant trends. Nearly 79.10% (1052/1330) of urine metabolic features and 74.58% (971/1302) of plasma metabolic features of borderline ovarian tumors were higher or lower than both benign and malignant ovarian tumors. This study is the first comprehensive description of changes in metabolites levels in urine and plasma between various ovarian tumors and changes in metabolite levels in urine and blood were consistent.

There are some previous metabolomic studies of ovarian cancer. Matthew F. Buas et al. performed a metabolomics analysis on plasma samples of 50 serous ovarian carcinoma and 50 serous benign controls. The AUC for Monte Carlo cross validation of the logistic regression model constructed with four lipid metabolites was 0.85, while the AUC of the hybrid model based CA125 and lipid metabolites reached 0.91 which was higher than 0.87 of CA125 alone [12]. Chen et al. identified and validated 27-nor-5b-cholestane-3,7,12,24,25 pentol glucuronide (CPG) in serum as diagnostic marker distinguishing EOC and benign samples, with AUC of 0.747 in validation set and AUC of 0.750 in stage I [19]. There were many other researches just looking for biomarkers between ovarian carcinoma and normal controls [7,11,20,21] or looking for biomarkers between ovarian carcinoma and benign ovarian tumors plus normal controls [17,22]. Yang et al. identified two potential serum biomarkers for diagnosing OC from normal controls, with high sensitivity (96.7%), specificity (66.7%) and AUC of 0.894 [7]. The AUC of these models were relatively high, probably because of the use of cross-validation rather than external validation or the intrinsic obvious difference between OC and normal controls. The clinical significance of this type of diagnostic model is not very large and according to our metabolomics results, the malignant tumors identified was likely to be benign tumors. Previous study using 21 early-stage EOC patients and 31 normal controls constructed an early diagnostic model based 18 plasma metabolites with an AUC of 0.897 without external validation, while the AUC of CA125 alone was 0.887 and the AUC of the hybrid model based on 18 metabolites and CA125 reached 0.935 [11]. Compared to these studies, our study had an adequate sample size and external validation set for more reliable results. Also, the number of candidate biomarkers was more likely to suitable for clinical application and the models had good classification performance. We also studied the difference between borderline ovarian tumors and OC. A previous study showed that analysis of differentiation between borderline ovarian tumors and OC showed an AUC of 0.824 for CA125 [23]. A previous article using 66 invasive ovarian carcinoma and 9 borderline ovarian tumors identified 51 differential metabolites from identified 291 metabolites. When verified by leave-one-out cross-validation, NMC and NCC classification model using all of the 291 metabolites had the best effect with accuracy of 87.9% and 88.9% [15]. Compared with previous study, our study had larger samples of borderline ovarian tumors (N = 21) and better classification performance with fewer metabolites (five putative urine metabolites, AUC: 0.943 and 0.836 in discovery and validation set respectively) and had external validation. But the borderline urine sample number in validation set is too low (N = 7), the results need further study.

Urinary concentrations of most fatty acyls, Stearic acid, “2,6 Dimethylheptanoyl carnitine,” Tetradecanal, Arachidic acid, Behenic acid, were decreased in ovarian tumor patients compared to normal controls. ”2,6 Dimethylheptanoyl carnitine” was also decreased in urine of bladder cancer compared to normal samples [24]. PS(46:1) which belongs to Phosphatidylserines, was down-regulated in ovarian tumor patients relative to that in normal controls, which further indicated the disturbance of the immune system in ovarian tumor patients. It has been suggested that the Phosphatidylserines is also profoundly dysregulated in the tumor microenvironment and antagonizes the development of tumor immunity [25]. The “1-(2,4,12-Octadecatrienoyl)piperidine” which belongs to the piperidine was also down-regulated in the urine of patients with ovarian tumors. Piperidine, through its unique scaffolds and excellent heterocyclic system, acts on the most important receptors and shows great potential in the field of anticancer drug development [26]. Ursolic acid and farnesol which exerts anticancer effects by inducing apoptosis [27,28] was decreased in benign ovarian tumors. This indicates that when the benign ovarian tumor has not yet developed into ovarian cancer, the body’s anti-cancer mechanism has begun to weaken. Ursolic acid was increased in OC compared to BOT, indicating the decreased apoptotic ability in malignant tumor environment.

Compared to BOT, OC had more dysregulated Steroid hormone biosynthesis pathway by urinary differential metabolites. Androsterone glucuronide and 16a-Hydroxyestrone involved in this pathway were down-regulated in urine of patients with OC. Compared with BOT, Steroids and steroid derivatives accounted for the largest proportion of urinary putative metabolites were downregulated in OC but these putative metabolites in BOT and OC were both lower than these in normal samples. This indicated that the steroids and steroid derivatives may be significant risk factor for ovarian disease status. Compared to normal controls, borderline and benign ovarian tumors, the urinary Cerulenin was down-regulated in OC, which is a fatty acid synthase inhibitor that induce tumor cell apoptosis [29]. Indoleacrylic acid was increased in borderline ovarian tumors compared with OC and BOT. A study showed that indoleacrylic acid production could promote anti-inflammatory responses and have therapeutic benefits [30]. It indicated that ovarian borderline tumors may have higher anti-inflammatory power than malignant and benign ovarian tumors. No functional information was retrieved in the articles or in the database for the other two putative metabolites (Coniferyl alcohol and (E)-Casimiroedine).

The overall level of plasma putative metabolites in OC relative to BOT was not consistent with urine. Most of differential metabolites were up-regulated in OC, especially the Lipids and lipid-like molecules. The 3,4-Dihydroxymandel which is a minor metabolite of norepinephrine in humans [31] was down-regulated in plasma of patients with OC compared to BOT. Patients with neuroblastoma experience elevated levels of 3,4-Dihydroxymandelic acid [31]. There is a possibility that ovarian cancer has a special mechanism associated with norepinephrine. “2-trans,4-cis-Decadienoylcarnitine” and 3-hydroxydodecanoyl carnitine which belongs to Acyl carnitines were up-regulated in plasma of patients with OC compared to BOT, while both of them were down-regulated in ovarian tumors compared to normal controls. Significantly increased plasma concentrations of Acyl carnitines was also detected in epithelia ovarian cancer patients compared with ovarian benign tumors in other study [14], while significantly decreased concentrations of Acyl carnitines was detected in early stage epithelia ovarian cancer patients compared to normal controls [11], which was consistent with our results. Acylcarnitine is involved in the fatty acid b-oxidation and the enhanced fatty acid b-oxidation in ovarian cancer compared to ovarian benign tumors was further confirmed [14]. Plasma 1,7-Dimethyl guanosine was evaluated in OC compared to BOT, which indicated the aberrant increase of tRNA methylases activity in OC than BOT [32]. No functional information was retrieved in the article or in the database for the other three putative metabolites (5′-O-Methylmelledonal, Tryptophyl-Tyrosine and Lucidenic acid A) associated with ovarian cancer.

Among the 10 differential plasma putative metabolites between malignant and borderline ovarian tumors, 8 putative metabolites were significantly higher or lower in borderline than malignant and benign ovarian tumors at the same time and 2 of 8 were also higher than normal controls. Elaidic acid with pro-metastasis effect [33] and Uridine triphosphate participating in energy support was down-regulated in borderline ovarian tumors compared to malignant and benign ovarian tumors. A previous study have also shown that Uridine was decreased in ovarian borderline tumors compared to ovarian carcinoma [15]. The above suggested that borderline ovarian tumors may have different molecular mechanisms from benign and malignant ovarian tumors.

Our study described comprehensive metabolomics changes in ovarian tumors with different degrees of urine and plasma samples for the first time. The overall trend of metabolite changes in different degrees of ovarian tumors in urine and plasma was found to be consistent. Our research also had some limitations. First, there are significant differences in age between different sample types but it also fully illustrates the age tendency of ovarian cancer. Second, a certain number of “unknown features” identified in our analysis which did not match the HMDB annotation information were not analyzed subsequently. Third, the sample size of borderline ovarian tumors was relatively small, which affects the accuracy analysis of the model. Fourth, HE4 and ROMA [23] are clinically referenced indicators. We did not compare our model with these two indicators because these two indicators were missing a lot in our clinical data.

## 4. Materials and Methods

### 4.1. Participants and Study Design

All subjects gave their informed consent for inclusion before they participated in the study. The study was conducted in accordance with the Declaration of Helsinki and the protocol was approved by Ethnic Committee of Fudan University Shanghai Cancer Center (approval code: 050432-4-1212B). A total of 420 patients with primary ovarian cancer (OC), benign ovarian tumor, borderline ovarian tumor and normal donors were prospectively enrolled from Fudan University Shanghai Cancer Center, Shanghai, China. Urine and plasma samples of participants were enrolled from September 2016 to May 2018 and were between the ages of 14 and 84 years. The enrolled patients did not receive any radiation, chemotherapy nor suffered from metabolic diseases including but not limited to liver diseases, diabetes and kidney diseases. In addition, patients who took drugs that proved to alter metabolism were also excluded. All ovarian carcinoma patients in this study underwent surgery and the diagnosis was further confirmed by at least two pathologists. The stages of OC were determined according to the International Federation of Gynecology and Obstetrics (FIGO) staging system [34]. The discovery set of our project consisted of 326 samples (plasma and urine: 40 normal controls, 36 benign, 13 borderline, 74 malignant, respectively. Among them, 148 participants had paired plasma and urine samples). An external cohort (plasma: 36 normal controls, 14 benign, 1 borderline, 21 malignant; urine: 40 normal controls, 45 benign, 7 borderline, 76 malignant) were employed as validation set.

Morning fasting blood were collected in a Vacutainer tube and the fasting first urine (normal controls were only fasting urine) were collected midstream in a 15 mL urine tube. The blood samples were kept at room temperature for 30 min for clotting. Clotted blood samples were centrifuged at a speed of 3000 rpm at 4 °C for 10 min to obtain the supernatant plasma and dispensed into a 1.5 mL centrifuge tube. Then they were quickly stored at −80 °C until the UHPLC-QTOF-MS analysis was conducted. Urine samples were temporarily stored at a 4 °C refrigerator within half an hour after collection. Then they were centrifuged at a speed of 4000 rpm at 4 °C for 10 min. Finally, the supernatant was transferred to 5 mL centrifuge tubes and stored at −80 °C.

### 4.2. HPLC-MS Analysis

#### 4.2.1. Sample Preparation

Samples of plasma and urine were prepared as reference [35] with slight modifications. In 10 mL polypropylene vials, 400 μL plasma or urine was added. Subsequently, 200 μL 5% (*v/v*) H3PO4 was added and vigorously vortexed for 30 s. Then, 4 mL salt solution and 4 mL organic solvent were added successively to samples and mixed 30 s after each step with a vortexer. The mixture was centrifuged 10 min (3000 rpm). The supernatant (4 mL) was transferred to 5 mL vials and dried under a steady stream of nitrogen. Dried sample was further treated with 1 mL isopropanol for desalting. After centrifugation, the supernatant was transferred to 1.5 mL vials and dried. For plasma the residue was dissolved in 50 µL of 15% acetonitrile with 0.2%FA and 30 µL dichloromethane (DCM), for urine the residue was dissolved in 80 µL of 15% acetonitrile with 0.2%FA and 40 µL DCM. Then centrifuged 40 min (15,000 rpm). The supernatant was transferred to clean vials for sample injection. The Lower-phase 15 µL was transferred to 1.5 mL vials and dried. The residue was dissolved in 50 µL of 60% acetonitrile with 0.2%FA and then 2µL was injected into UHPLC-QTOF system for analysis. To monitor the robustness of sample preparation and the stability of instrument analysis, we prepared quality control (QC) sample by pooling equal aliquots of plasma/urine from 20% random screening samples. The pre-treatment of QC samples was consistent with real samples.

#### 4.2.2. Liquid Chromatography-Mass Spectrometry

Metabolic profiling of plasma polar sample (PU), plasma nonpolar sample (PL), urine polar sample (UU), urine nonpolar sample (UL) in a discovery set and validation set based on nontargeted analysis was collected by an Agilent 1290 Infinity LC system coupled to an Agilent G6200 Series time-of-flight mass spectrometer (Agilent, USA). An Agilent Poroshell 120 EC-C18 (50 mm × 2.1 mm, 2.7 μm) column was used for chromatographic separation. The mobile phase was composed of solvent A, 0.5% FA and solvent B, acetonitrile. The gradient applied for Upper-phase was as follows: 0–5 min, 2–20% B, 5–9 min, 20–80% B, 9–10 min, 80–100%B, 10–12.5 min, 100%B, 12.5–15 min, 2%B at a flow rate of 0.3 mL/min with a sample volume of 5 μL. The gradient applied for Lower-phase was as follows: 0–4 min, 20–70% B, 4–10 min, 70–100% B, 10–12.5 min, 100%B, 12.5–15 min, 20%B at a flow rate of 0.3 mL/min with a sample volume of 2 μL. Electrospray ionization mass spectrometry (ESI-MS) experiment source conditions were as follows: nebulizer pressure, 40 psi; drying gas, 9.0 L/min; and gas temperature, 350 °C. Capillary voltage was set to 3.5 kV and the skimmer voltage was 65 V. The mass scan rate was 100–1500 *m*/*z* for Upper-phase and 100–1200 *m*/*z* for Lower-phase and the data were collected with Agilent MassHunter Workstation Software.

The disease samples and normal control samples in the first and second batches were alternated with respect to run order to avoid batch effects. Moreover, QC samples were inserted into the analytical sequence after each set of 20 real samples.

### 4.3. Data Statistics and Analysis

The raw data acquired by UHPLC-QTOF in positive ion mode were imported to XCMS to pre-treat data including chromatographic peak extraction, peak matching, retention time correction and filling of missing values. The resulting data consisted of retention time, M/Z value and normalized peak area. Further data pre-processing on two batches of samples was carried out using R-Studio software (R-3.5.3). Variables missed in 20% or greater of the samples (“80% rule”) or coefficient of variation greater than 30% in QC samples were removed from further statistical analysis and the missing values (i.e., zeros) were replaced by 1/2 minimum [36]. Median normalization was then performed to bring the sample to the same level and reduce the batch effect. Log2 transform was performed to adjust data distribution. An unsupervised model of principal component analysis (PCA) with unit variance scaling was applied in two batches of samples using processed data to assess the overall metabolome alterations among groups and monitor the stability of the study. Each batch contained 4 sample types and a portion of plasma and urine paired samples. In order to reduce the impact of sample collection time on subsequent analyses and considering that half of the samples were plasma and urine paired samples, the plasma and urine paired samples were selected as the discovery set and the remaining samples as the validation set. After splitting the data to discovery and validation sets, the orthogonal partial least-squared discriminant analysis (OPLS-DA) with pareto scaling was performed in discovery set to maximize the distance between groups and identify important variables with an important contribution to the classification according to its variable important in the projection (VIP). A permutation test was performed 1000 times to access the risk of overfitting for the model.

Univariate and multivariate analysis was performed using the R-Studio software (R-3.5.3). A Wilcoxon Mann-Whitney test with Benjamin-Hochberg-based false discovery rate (FDR) was used for the statistical analysis. VIP > 1 and FDR < 0.1 were the threshold for differential putative metabolites between normal controls and benign ovarian tumors (BOT), borderline ovarian tumors and ovarian carcinoma (OC). VIP > 1 and *p*-value < 0.05 were the thresholds for differential putative metabolites between BOT, borderline ovarian tumors and OC. Subsequently, the primary and secondary mass spectrometry data of the differential putative metabolites were imported into the Progenesis QI (Waters) software and the HMDB database (http://www.hmdb.ca) was searched. The mass deviations of precursor ions and product ions are set to 20 ppm and 100 ppm, respectively. The search result score of precursor ions is >40 and the fragment ion score is >30, which is regarded as the final putative identification. After further pareto scaling on the processed data above, Support Vector Machine (SVM) with radial kernel was used to build the model based on the differential putative metabolites, considering that the SVM algorithm works well for small samples, can solve the problems of high-dimensionality and nonlinear feature interactions and has strong generalization ability. Considering the clinical application and model effect, the exhaustive method and 7-fold cross-validation were used to select the best classification model within 10 variables. A receive-operating characteristic (ROC) curve was used to evaluate the results of the regression analysis. The categories of differential putative metabolites were searched through HMDB (http://www.hmdb.ca).

## 5. Conclusions

In summary, our study described the urinary and plasma metabolomic profile of different types of ovarian tumors. Our results demonstrated the potential usage of urine and plasma metabolic strategies for the diagnosis of different types of ovarian tumors. A capable model which can help in early-diagnosis of ovarian cancer was also constructed.

## Figures and Tables

**Table 1:** Clinic characteristics of the samples in each set.

	Discovery Set				Validation Set							
	Plasma/Urine				Plasma				Urine			
Level	Normal	Benignant	Borderline	Malignant	Normal	Benignant	Borderline	Malignant	Normal	Benignant	Borderline	Malignant
n	40	36	13	74	36	14	1	21	40	45	7	76
Age (mean (sd))	39.67 (8.50)	43.33 (14.55)	46.54 (17.59)	56.19 (9.77)	40.83 (10.17)	44.36 (17.08)	34	54.76 (11.84)	45.85 (9.72)	43.51 (14.81)	38.86 (13.87)	53.75 (10.00)
CA125 (mean (sd))	8.35 (5.81)	83.45 (234.82)	183.88 (326.63)	1254.34 (1597.27)	6.31 (3.78)	60.96 (146.43)	250.2	938.10 (1243.80)	7.65 (6.12)	94.57 (321.85)	193.12 (140.65)	1078.35 (1195.30)
HE4 (mean (sd))	-	53.71 (13.77)	84.34 (66.89)	440.62 (447.08)	-	52.28 (10.99)	74.06	389.75 (472.48)	-	49.93 (23.99)	93.45 (40.62)	519.58 (405.72)
FIGO (%)	-	-	-	-	-	-	-		-	-	-	-
I	-	-	8 (61.5)	8 (10.8)	-	-	-	2 (9.5)	-	-	6 (85.7)	13 (17.1)
II	-	-	1 (7.7)	9 (12.2)	-	-	1 (100.0)	3 (14.3)	-	-	-	7 (9.2)
III	-	-	2 (15.4)	50 (67.6)	-	-	-	15 (71.4)	-	-	1 (14.3)	51 (67.1)
IV	-	-	-	7 (9.5)	-	-	-	-	-	-	-	5 (6.6)
NA	40 (100.0)	36 (100.0)	2 (15.4)	-	36 (100.0)	14 (100.0)	-	1 (4.8)	40 (100.0)	45 (100.0)	-	-
Pathology (%)												
HGSOC	-	-	-	57 (77.0)	-	-	-	14 (70.0)	-	-	-	56 (73.7)
OCS	-	-	-	1 (1.4)	-	-	-	3 (15.0)	-	-	-	3 (3.9)
OCCC	-	-	-	3 (4.1)	-	-	-	2 (10.0)	-	-	-	4 (5.3)
OEC	-	-	-	-	-	-	-	-	-	-	-	4 (5.3)
Others	-	-	-	13 (17.5)	-	-	-	2 (10.0)	-	-	-	9 (11.8)

Abbreviations: HGSOC: High-grade serous ovarian cancer; OCS: ovarian carcinosarcoma; OCCC: ovarian clear cell carcinoma; OEC: ovarian endometrioid adeno-carcinoma.

**Table 2:** The information of biomarker panels.

Compound_Name	RT (min)	*m/z*	Delta (ppm)	*p*-Value	FDR	log2FC	VIP	Super Class	Class	Direct Parent
**Normal vs. Ovarian tumor (Urine)**										
1-(2,4,12-Octadecatrienoyl) piperidine	3.2758	363.329	5.47	7.16 × 10^−23^	5.17 × 10^−22^	−2.7274	1.2971	Organoheterocyclic compounds	Piperidines	N-acylpiperidines
PS(46:1)	5.7625	477.3462	−0.77	1.19 × 10^−25^	1.97 × 10^−24^	−5.9980	1.8726	Lipids and lipid-like molecules	Glycerophospholipids	Phosphatidylserines
**Benign vs. Malignant (borderline + malignant) (Plasma)**										
5′-O-Methylmelledonal	1.5925	485.158	3.19	0.0248	0.3291	−0.3308	1.4676	Lipids and lipid-like molecules	Prenol lipids	Melleolides and analogues
Tryptophyl-Tyrosine	2.3507	409.1868	0.15	0.0146	0.2873	−0.2541	2.0151	Organic acids and derivatives	Carboxylic acids and derivatives	Dipeptides
3,4-Dihydroxymandelic acid	3.7132	185.0443	0.29	0.0360	0.3513	−0.5068	1.5459	Benzenoids	Phenols	Catechols
Lucidenic acid A	4.9003	476.306	12.25	0.0139	0.2868	1.1363	1.4422	Lipids and lipid-like molecules	Prenol lipids	Triterpenoids
2-trans,4-cis-Decadienoyl carnitine	7.0519	312.2159	0.45	0.0126	0.2780	0.4928	1.1053	Lipids and lipid-like molecules	Fatty Acyls	0
**Borderline vs. Malignant (Urine)**										
16a-Hydroxyestrone	5.0385	269.1495	−13.21	0.0127	0.6276	−0.7163	2.1758	Lipids and lipid-like molecules	Steroids and steroid derivatives	Estrogens and derivatives
Coniferyl alcohol	5.2862	181.0855	4.04	0.0123	0.6276	−0.7280	2.2225	Benzenoids	Phenols	Methoxyphenols
Indoleacrylic acid	5.4391	171.0637	3.20	0.0286	0.6276	−0.4444	1.5046	Organoheterocyclic compounds	Indoles and derivatives	Indoles
(E)-Casimiroedine	6.0093	436.2202	−1.56	0.0008	0.6276	−0.5977	2.1053	Phenylpropanoids and polyketides	Cinnamic acids and derivatives	Glycosylamines
Cerulenin	7.2691	224.1282	6.21	0.0132	0.6276	−0.5413	1.6339	Organoheterocyclic compounds	Epoxides	Oxirane carboxylic acids and derivatives

RT: Retention Time; *m/z*: Mass-to-Charge Ratio; Delta: the mass accuracy to the theoretical *m/z* and the detected ion; log2FC: log2 Fold Change.

**Table 3:** Results of measurement of the putative metabolite panel, CA125 or Both in the diagnosis of ovarian tumor or ovarian cancer.

Heading	Discovery Set				Validation Set		
	AUC	Cutoff	Sensitivity	Specificity	AUC	Sensitivity	Specificity
**Normal vs. Ovarian tumor (Urine)**							
2-Meta	0.987	0.855	94.26%	95.00%	0.984	97.66%	87.50%
CA125	0.965	14.78	87.70%	95.00%	0.978	89.84%	95.00%
**Benign vs. Malignant (borderline + malignant) (Plasma)**							
5-Meta	0.876	0.72	87.36%	62.86%	0.896	86.36%	78.57%
CA125	0.882	35	88.51%	65.71%	0.903	86.36%	71.43%
5-Meta + CA125	0.972	0.71	93.10%	91.43%	0.932	90.91%	92.86%
**Borderline vs. Malignant (Urine)**							
5-Meta	0.943	0.828	98.65%	84.62%	0.836	80.26%	71.43%
CA125	0.830	165.5	81.08%	76.92%	0.807	80.26%	42.86%

Abbreviations: 2-Meta, panel of two putative metabolite; 5-Meta, panel of five putative metabolite.

**Table 4:** Results of measurement of the plasma putative metabolite panel, CA125 or Both in the diagnosis of early-stage ovarian cancer from benign ovarian tumor.

Heading	Discovery Set				Validation Set		
	AUC	Cutoff	Sensitivity	Specificity	AUC	Sensitivity	Specificity
**Benign vs. Malignant (borderline + malignant) (Plasma)**							
5-Meta	0.847	0.72	80.77%	62.86%	0.988	100%	78.57%
CA125	0.733	35	69.23%	65.71%	0.893	83.33%	71.43%
5-Meta + CA125	0.922	0.71	80.77%	91.43%	1	100%	92.86%

Abbreviations: 5-Meta, plasma putative metabolite panel.

## Supplementary Materials

The following are available online at https://www.mdpi.com/2072-6694/12/12/3642/s1, Figure S1: Technique pipeline to identifying potentially biomarkers panels in three comparison groups, Figure S2: Score plots of PCA from the two batch of urine samples, Figure S3: Score plots of PCA from the two batch of plasma samples, Figure S4: The OPLS-DA score plot and the permutation test results of urine samples, Figure S5: The OPLS-DA score plot and the permutation test results of plasma samples, Figure S6: Compound category of differential putative metabolites, Figure S7: Prediction plot of the hybrid model for distinguishing benign and malignant (borderline + malignant) ovarian tumors, Table S1. Other clinical characteristics of the samples in each set. Table S2: Statistical analysis of differential urine putative metabolites from three group of comparison, Table S3: Statistical analysis of differential plasma putative metabolites from three group of comparison.

## Abbreviations

OC	Ovarian Cancer
BOT	Benign Ovarian Tumor
UHPLC-QTOF-MS	Ultra-High-Performance Liquid Chromatography Quadrupole Time-of-flight Mass Spectrometry
CA125	Cancer Antigen 125
HE4	Human Epididymis Protein 4
QC	Quality Control
PCA	Principal Component Analysis
OPLS-DA	Orthogonal Partial Least-Squared Discriminant Analysis
PLS-DA	Partial Least-Squared Discriminant Analysis
VIP	Variable important in the Projection
FDR	False Discovery Rate
SVM	Support Vector Machine
ROC	Receiver Operating Characteristic Curve
AUC	Area Under Curve
PS	Phosphatidylserine
PU	plasma polar sample
PL	plasma nonpolar sample (PL)
UU	urine polar sample
UL	urine nonpolar sample
