# Supplementary material for: Untargeted Metabolomic Characterization of Ovarian Tumors
Source: Cancers (Basel). 2020 Dec 4;12(12):3642. doi: 10.3390/cancers12123642 (PMC7761955; doi:10.3390/cancers12123642)
Supplement: Supplementary file 1 [file cancers-12-03642-s001.pdf]

cancers

Supplementary material

Untargeted Metabolomic Characterization of Ovarian Tumors

Xiaona Liu, Gang Liu, Lihua Chen, Fei Liu, Xiaozhe Zhang, Dan Liu, Xinxin Liu, Xi Cheng and Lei Liu

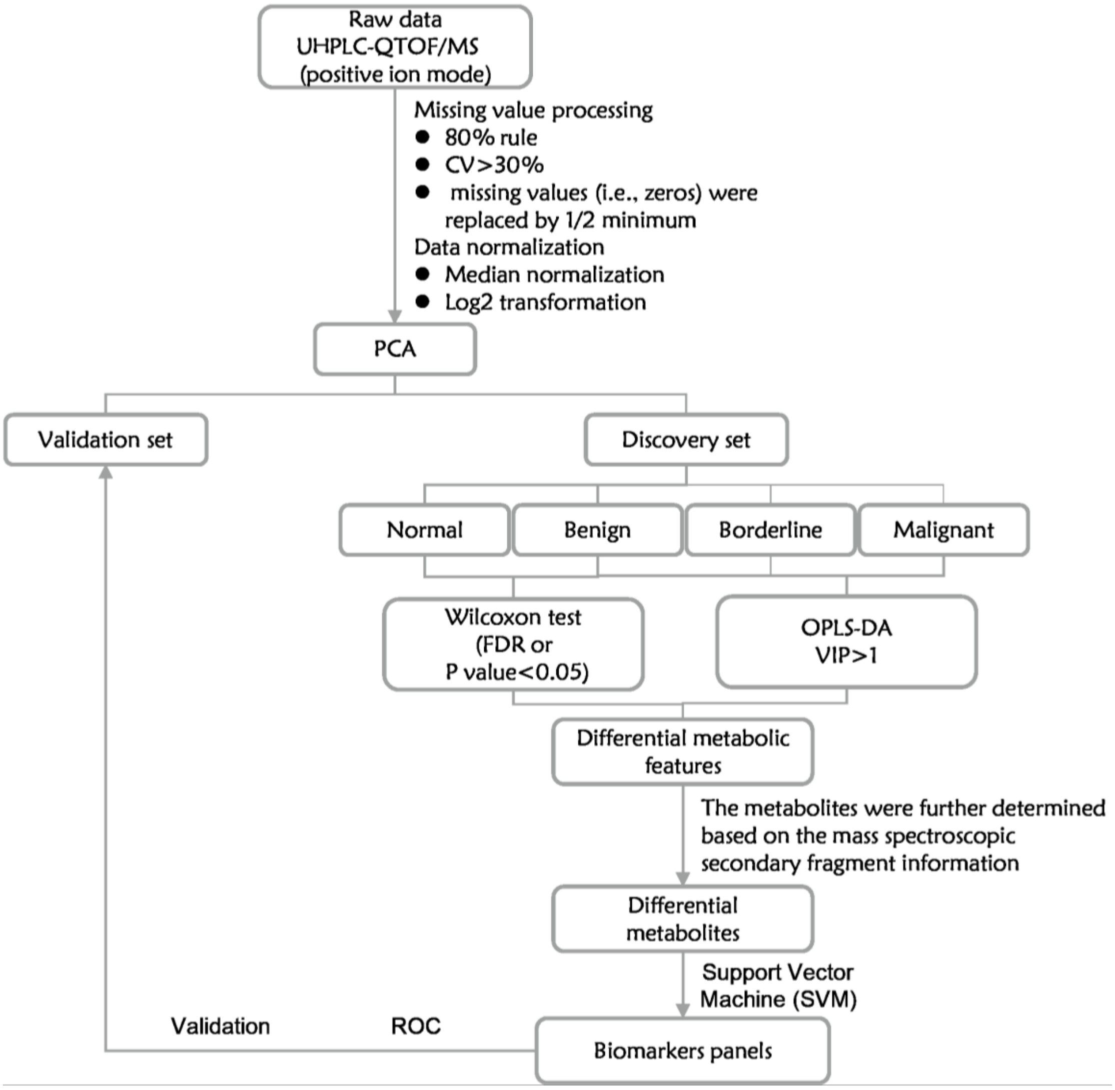

Figure S1. Technique pipeline to identifying potentially biomarkers panels in three comparison groups.

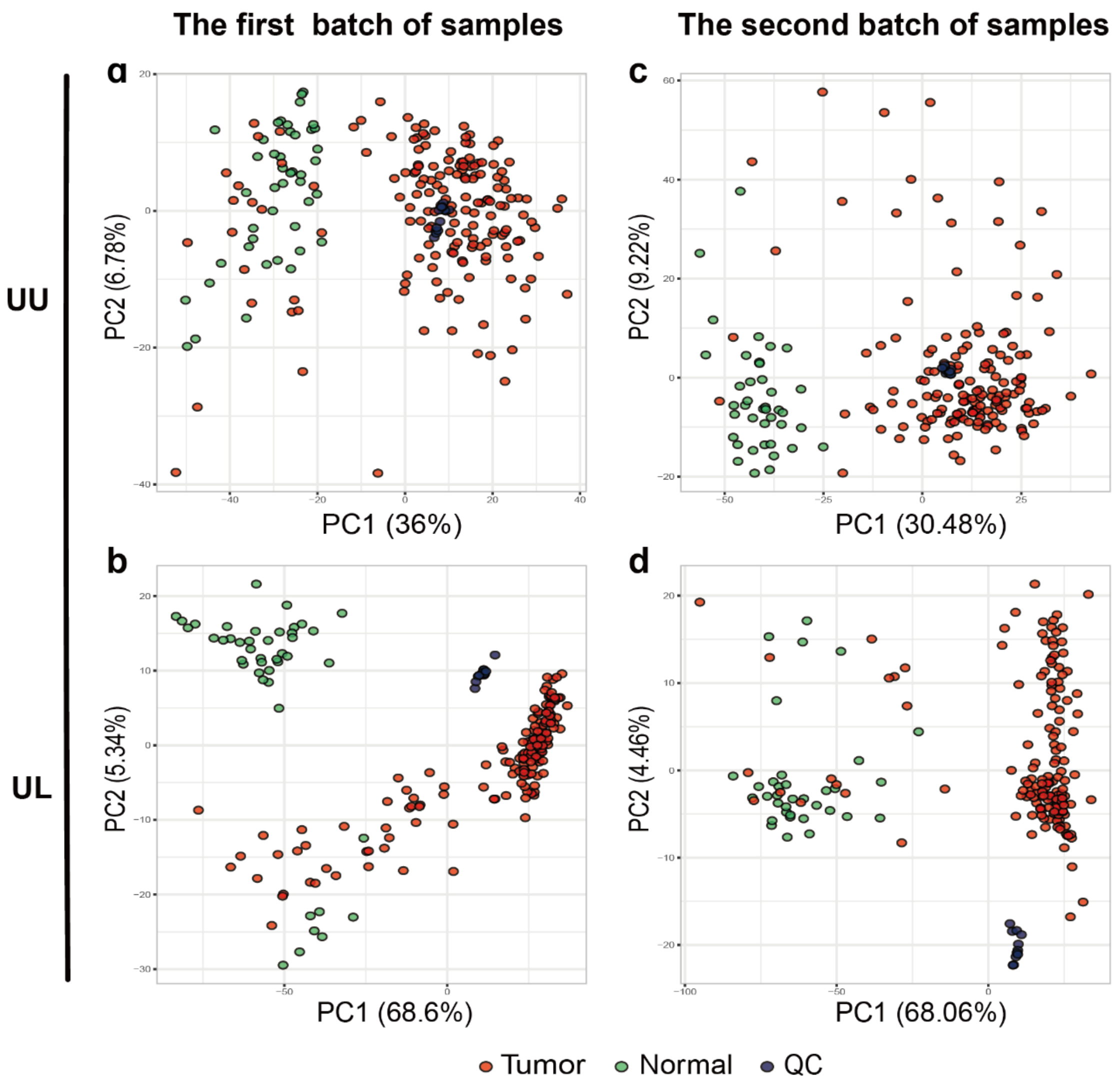

Figure S2. Score plots of PCA from the two batch of urine samples. (**a, b**) PCA score plots of UU, and UL from the first batch of urine samples. (**c, d**) PCA score plots of UU, and UL from the second batch of urine samples. UU, urine polar sample; UL, urine nonpolar sample. Red plots indicate ovarian tumors. Green plots indicate normal controls. Blue plots indicate quality control samples.

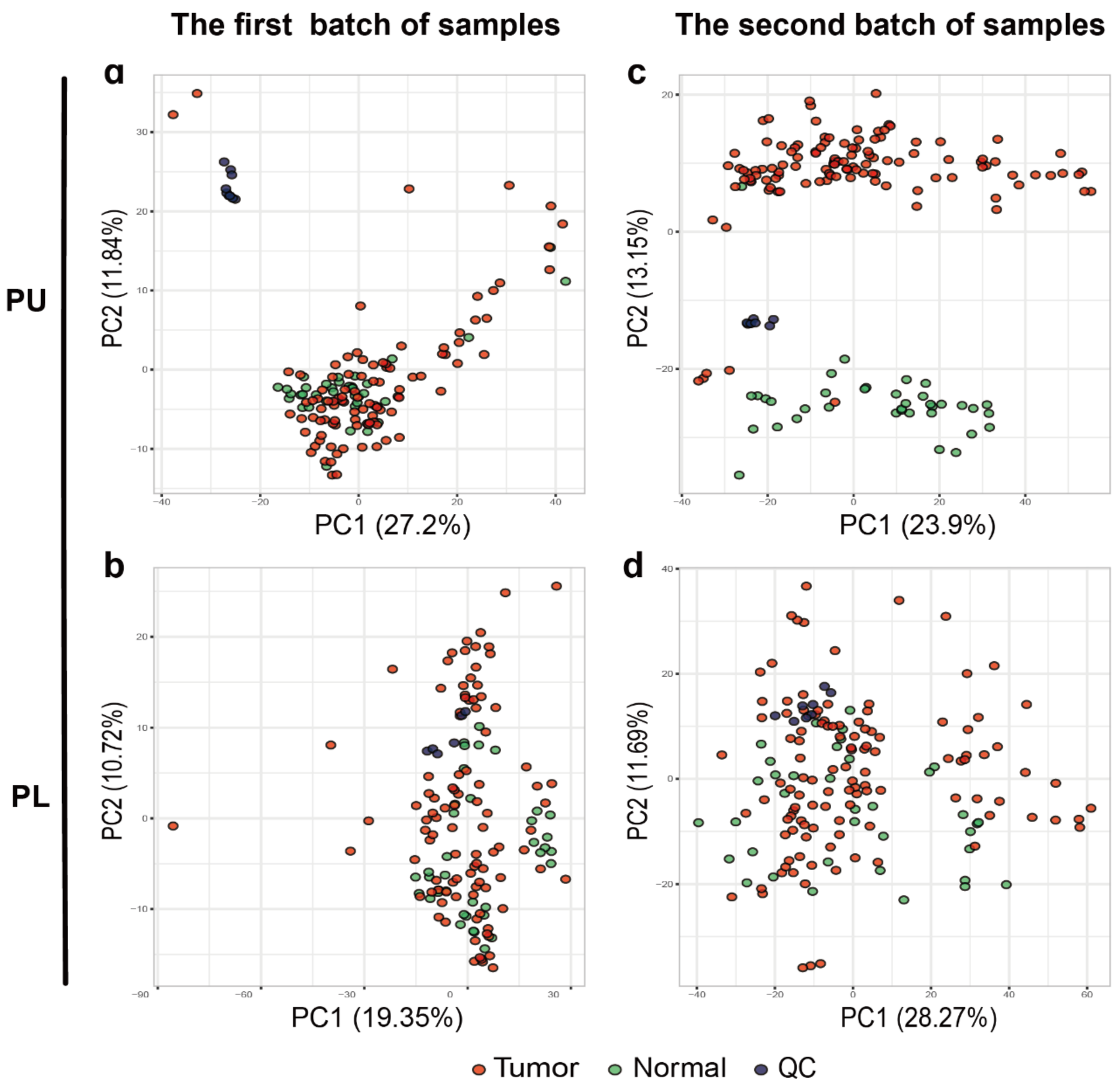

Figure S3. Score plots of PCA from the two batch of plasma samples. (**a, b**) PCA score plots of PU, and PL from the first batch of plasma samples. (**c, d**) PCA score plots of PU, and PL from the second batch of plasma samples. PU, plasma polar sample; PL, plasma nonpolar sample. Red plots indicate ovarian tumors. Green plots indicate normal controls. Blue plots indicate quality control samples.

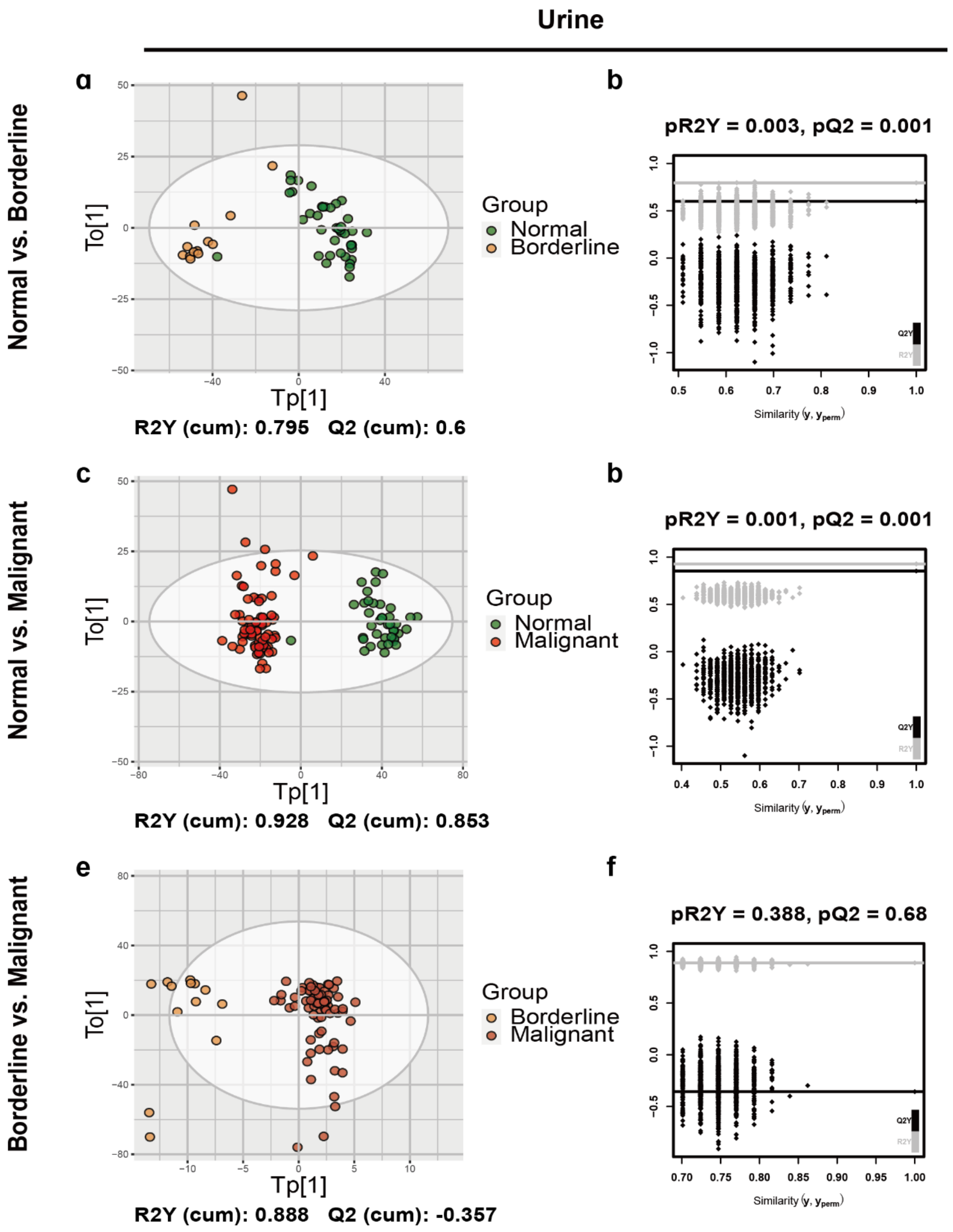

Figure S4. The OPLS-DA score plot and the permutation test results of urine samples. The OPLS-DA score plot between (**a**) normal controls and borderline, (**c**) normal with malignant, (**e**) borderline and malignant ovarian tumors in urine samples, and the corresponding permutation test results (**b**), (**d**), (**f**).

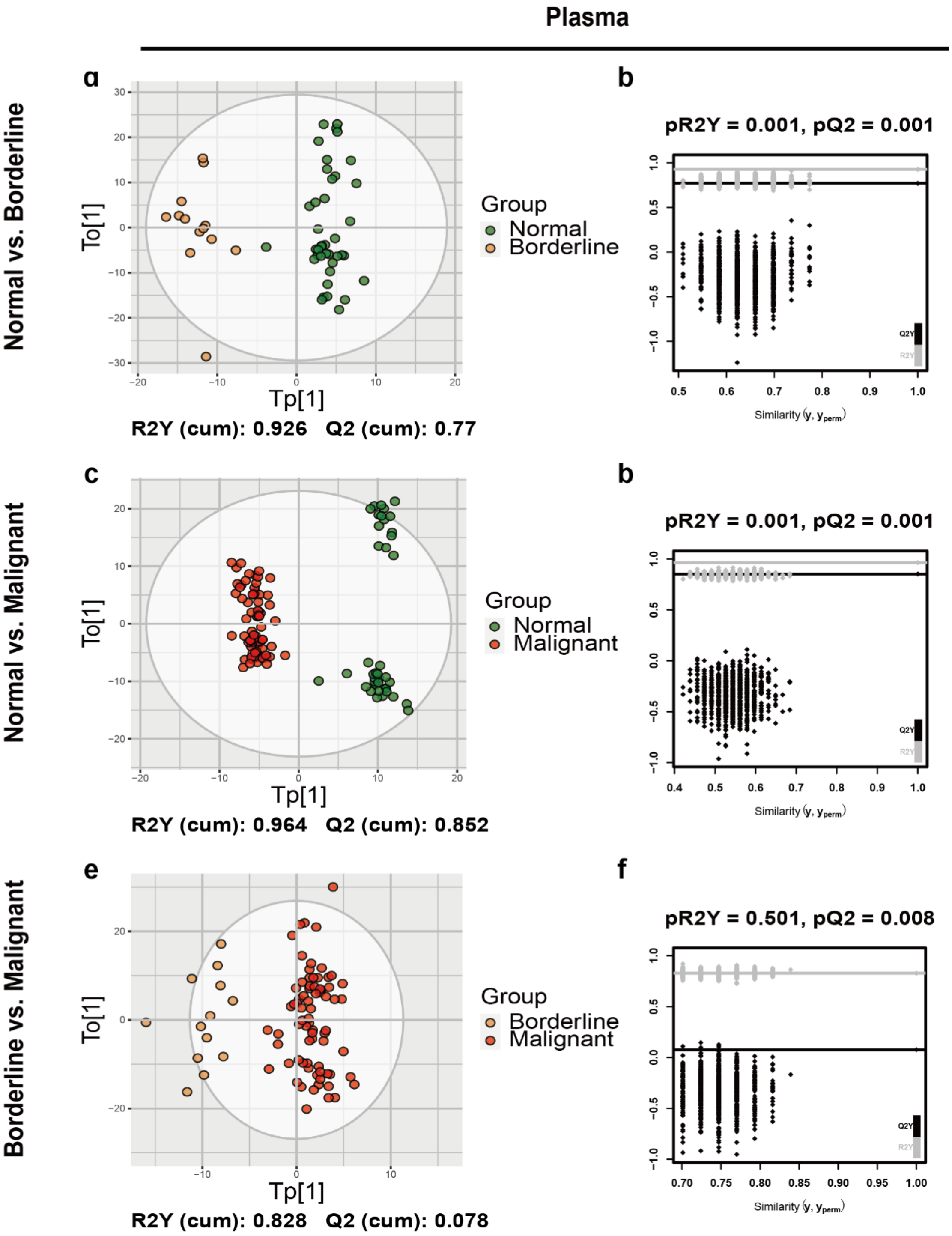

Figure S5. The OPLS-DA score plot and the permutation test results of plasma samples. The OPLS-DA score plot between (**a**) normal controls and borderline, (**c**) normal and malignant, (**e**) borderline and malignant ovarian tumors in plasma samples, and the corresponding permutation test results (**b**), (**d**), (**f**).

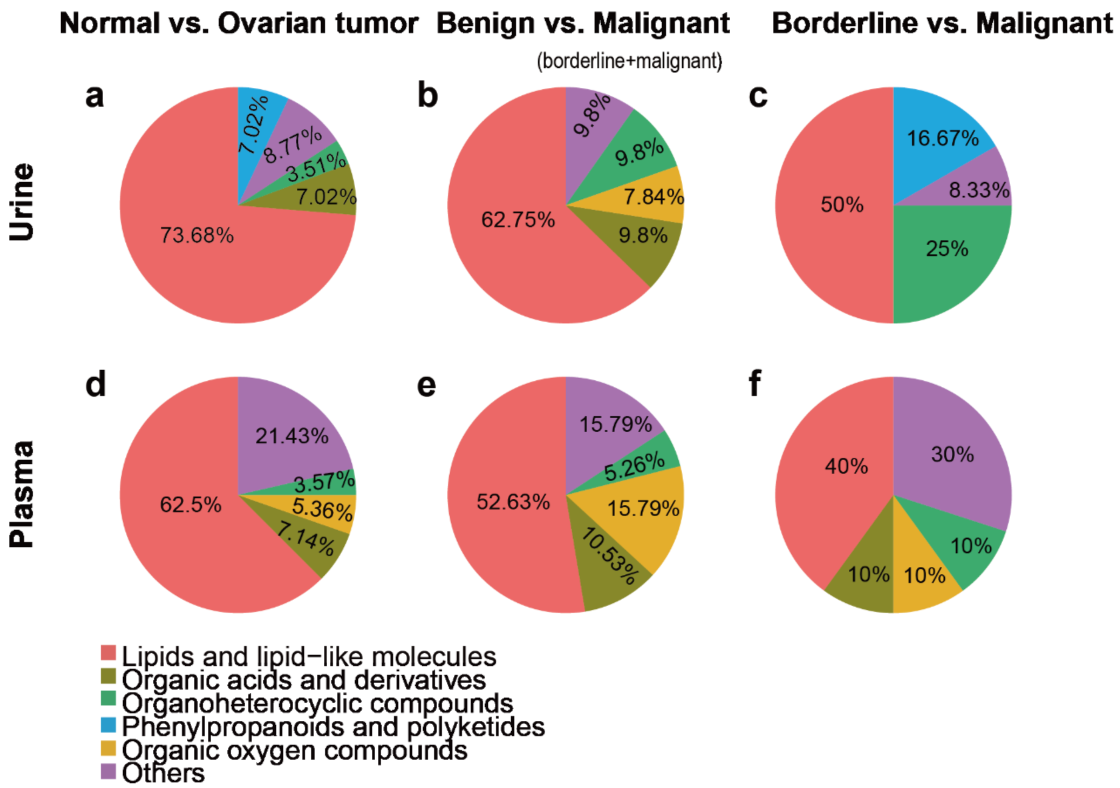

Figure S6. Compound category of differential metabolites. Compound category of differential metabolites in urine between (**a**) normal controls and ovarian tumors, (**b**) benign and malignant (borderline+malignant), and (**c**) borderline and malignant ovarian tumors. Compound category of differential metabolites in plasma between (**d**) normal controls and ovarian tumors, (**e**) benign and malignant (borderline+malignant), and (**f**) borderline and malignant ovarian tumors.

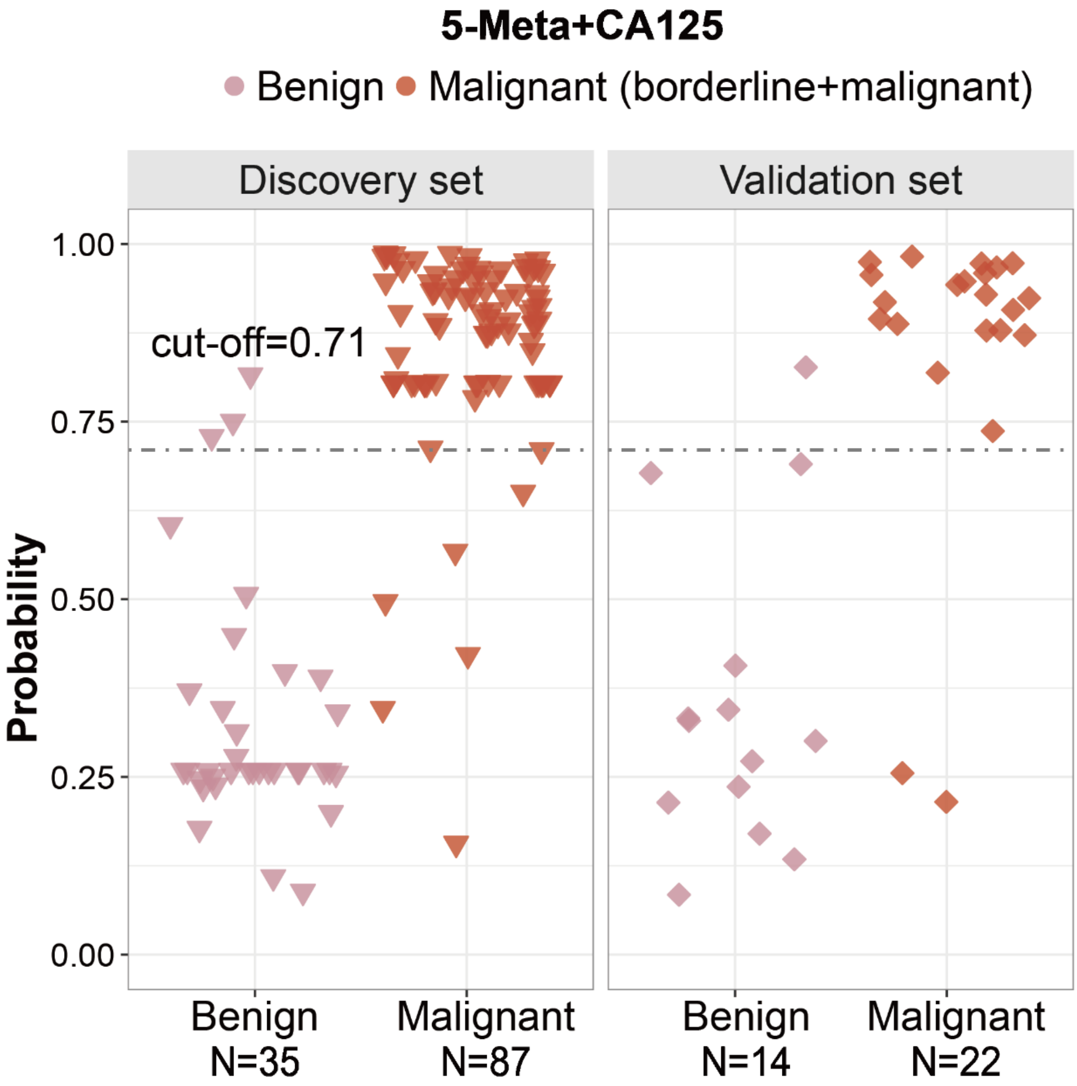

Figure S7. Prediction plot of the hybrid model for distinguishing benign and malignant (borderline+malignant) ovarian tumors. The prediction performance by the hybrid based on 5 plasma metabolites and CA125 for diagnosing malignant (borderline+malignant) ovarian tumors from benign ovarian tumors in the discovery and validation set.

Table S1. Other clinical characteristics of the samples in each set.

.	Discovery set				Validation set							
	Plasma/Urine				Plasma				Urine			
level	Normal	Benignant	Borderline	Malignant	Normal	Benignant	Borderline	Malignant	Normal	Benignant	Borderline	Malignant
n	40	36	13	74	36	14	1	21	40	45	7	76
**CA199 (mean (sd))**	16.89 (10.60)	52.12 (169.76)	65.96 (132.63)	74.35 (218.29)	15.18 (9.08)	25.64 (37.42)	10.08	49.41 (102.10)	15.01 (8.57)	68.48 (143.49)	113.47 (151.48)	45.89 (128.19)
**AFP (mean (sd))**	4.12 (1.54)	2.63 (1.35)	3.09 (1.27)	55.15 (433.60)	3.69 (0.97)	2.56 (1.34)	1.16	22.97 (74.33)	3.65 (1.20)	2.65 (1.26)	3.55 (2.24)	3.77 (5.70)
**CEA (mean (sd))**	1.73 (0.53)	1.30 (0.70)	1.89 (1.46)	4.60 (12.55)	1.77 (0.49)	1.39 (0.81)	0.19	9.69 (26.79)	1.76 (0.59)	1.46 (0.88)	1.88 (1.20)	6.49 (30.05)
**SCCA (mean (sd))**	0.68 (0.55)	0.76 (0.28)	0.68 (0.26)	1.39 (3.83)	0.66 (0.29)	0.84 (0.23)	0.3	0.65 (0.36)	0.61 (0.30)	0.80 (0.43)	0.78 (0.30)	0.77 (0.58)
**NSE (mean (sd))**	5.88 (2.16)	10.37 (2.27)	12.11 (1.93)	20.68 (15.26)	4.73 (1.05)	10.25 (2.09)	11.75	15.80 (6.13)	5.28 (1.38)	10.49 (2.29)	9.94 (0.67)	17.70 (9.31)

Table S2. Statistical analysis of differential urine metabolites from three group of comparison.

Metabolites	RT (min)	*m/z*	Delta (ppm)	*P*-value	FDR	log2FC	VIP
**Normal vs. Ovarian tumor**							
2,2,6,7-Tetramethylbicyclo[4.3.0]nona-1(9),4-diene-7,8-diol	2.4702	226.1787	−18.25	7.21×10^{-14}	1.33×10^{-13}	−1.7684	1.0488
HMDB0126628	2.9224	576.1293	1.75	1.19×10^{-19}	4.99×10^{-19}	−2.4636	1.2012
Feruloylcholine	4.6002	298.1857	−9.69	1.37×10^{-12}	2.34×10^{-12}	2.4574	1.0582
9'-Carboxy-gamma-tocotrienol	3.5418	391.2703	−4.21	1.10×10^{-13}	2.01×10^{-13}	−2.1683	1.0421
6-Keto-prostaglandin F1a	4.0455	409.1832	−18.84	4.50×10^{-14}	8.45×10^{-14}	1.6085	1.0877
Austalide L	4.5637	448.2644	2.88	1.14×10^{-14}	2.26×10^{-14}	2.2649	1.0739
Capsoside A	6.2219	717.3676	−0.06	5.17×10^{-15}	1.06×10^{-14}	2.5178	1.0142
Quinine	5.1454	325.1856	−14.12	1.77×10^{-15}	3.76×10^{-15}	2.6304	1.2532
Ginsenoyne C	4.9177	277.1789	1.26	6.42×10^{-16}	1.42×10^{-15}	−1.5775	1.0796
Porphobilinogen	4.4608	227.6054	1.32	4.48×10^{-16}	1.01×10^{-15}	2.1794	1.1076
Ursolic acid	7.3843	495.3321	14.06	4.75×10^{-17}	1.21×10^{-16}	−1.6310	1.0472
3-Oxocholic acid	7.281	407.2759	4.85	4.52×10^{-17}	1.15×10^{-16}	−1.5103	1.0353
PE(36:4)	5.5261	722.5014	−8.56	3.55×10^{-17}	9.13×10^{-17}	−3.8321	1.3991
Thromboxane	5.0003	316.3472	0.95	3.07×10^{-17}	8.01×10^{-17}	−1.5745	1.0652
(Z)-8-Tetradec enal	5.3471	228.2314	10.53	2.92×10^{-17}	7.68×10^{-17}	−1.3385	1.0096
Behenic acid	4.9745	358.3667	0.59	2.78×10^{-17}	7.32×10^{-17}	−1.3734	1.0363
Farnesol	9.1546	240.2321	0.93	2.17×10^{-17}	5.79×10^{-17}	−1.5129	1.0080
beta-Elemonic acid	7.8083	477.3189	−27.98	1.97×10^{-17}	5.29×10^{-17}	−1.4318	1.0059
Dihydrocumambrin A	4.6367	347.1247	0.36	1.78×10^{-17}	4.87×10^{-17}	−1.6317	1.0522
(Z)-3-Dodecene	3.2438	186.2208	0.81	1.46×10^{-17}	4.05×10^{-17}	−1.4175	1.0762
DAG(18:0)	5.2985	395.2757	1.83	1.08×10^{-17}	3.06×10^{-17}	−1.4892	1.0204
Pubescenol	7.1438	475.2956	−10.33	7.59×10^{-18}	2.21×10^{-17}	−1.5887	1.0640
Geranylacetone	9.1587	212.2008	6.52	5.88×10^{-18}	1.74×10^{-17}	−1.4734	1.0024
13'-Carboxy-gamma-tocopherol	6.0073	429.317	−22.08	3.89×10^{-18}	1.19×10^{-17}	−2.3616	1.2038
(ent-16alpha)-16-Kauranol	7.0928	308.2937	0.06	3.89×10^{-18}	1.19×10^{-17}	−1.6052	1.0410
LPA(13:0)	5.6308	407.1677	22.05	1.51×10^{-18}	5.04×10^{-18}	2.7261	1.2605
Poly sorbate 60	4.3144	474.2543	1.24	1.22×10^{-18}	4.11×10^{-18}	−1.7537	1.0325
PG(42:9)	6.4607	423.7773	11.28	1.22×10^{-18}	4.11×10^{-18}	2.5956	1.0377
Geranylcitronellol	7.9792	310.3101	5.97	1.15×10^{-18}	3.93×10^{-18}	−1.5769	1.0344

Lucidenic acid A	4.8555	497.2351	13.97	6.36×10^{-19}	2.26×10^{-18}	2.3894	1.2931
Hypoglycin B	5.2024	541.2619	24.80	3.47×10^{-19}	1.31×10^{-18}	2.4610	1.2619
1,28-Octacosanediol diferulate	6.3904	401.7641	−14.89	2.77×10^{-19}	1.08×10^{-18}	2.6515	1.0372
Arachidic acid	4.4395	332.3389	6.94	1.77×10^{-19}	7.12×10^{-19}	−1.6119	1.0941
Normetanephrine	4.0742	184.0977	5.19	1.67×10^{-19}	6.77×10^{-19}	−2.8846	1.2534
Opiorphin	6.2559	710.417	17.16	1.58×10^{-19}	6.46×10^{-19}	2.9139	1.2039
PG(36:1)	7.7177	800.5676	18.28	7.47×10^{-20}	3.27×10^{-19}	−3.9998	1.6180
Tetradecanal	3.2267	231.2506	8.82	6.64×10^{-20}	2.96×10^{-19}	−1.8185	1.1799
LPC(18:2)	5.1949	521.3356	−13.08	5.91×10^{-20}	2.67×10^{-19}	2.8174	1.3272
Cynarasaponin F	6.4343	798.4699	7.74	5.91×10^{-20}	2.67×10^{-19}	2.8149	1.1725
[6]-Dehydrogingerdione	6.6439	308.1856	−4.05	3.69×10^{-20}	1.72×10^{-19}	−2.8805	1.0336
3-trans-p-Coumaroylrotundic acid	6.0411	652.4109	−11.75	2.58×10^{-20}	1.22×10^{-19}	3.2086	1.1423
Stearic acid	3.8492	303.3076	4.01	2.29×10^{-20}	1.10×10^{-19}	−2.0975	1.1505
Aspartyl-Glycine	4.5817	208.0968	21.15	1.91×10^{-20}	9.33×10^{-20}	−1.8588	1.0535
9,12,13-TriHOME	4.9037	313.234	−2.97	1.69×10^{-20}	8.41×10^{-20}	−1.7072	1.1424
PE(40:10)	6.3919	392.7493	−2.54	1.04×10^{-20}	5.28×10^{-20}	2.6696	1.0748
N-Methylnicotinium	3.2103	216.1033	6.94	4.05×10^{-21}	2.24×10^{-20}	−2.7077	1.2857
Muzanzagenin	6.2002	460.3105	15.30	2.77×10^{-21}	1.59×10^{-20}	4.1416	1.5427
PI(34:1)	6.4964	430.7669	−10.99	1.45×10^{-21}	9.00×10^{-21}	2.8262	1.1002
Ilicifolinoside A	3.1704	267.1252	−3.45	6.55×10^{-22}	4.19×10^{-21}	−1.7629	1.0448
Lc3Cer	6.5641	886.5224	11.68	2.53×10^{-22}	1.68×10^{-21}	2.7175	1.1140
1-(2,4,12-Octadecatrienoyl)piperidine	3.2758	363.329	5.47	7.16×10^{-23}	5.17×10^{-22}	−2.7274	1.2971
Hebevinoside V	8.389	873.5137	10.86	4.32×10^{-23}	3.25×10^{-22}	−2.5001	1.1237
2,6 Dimethylheptanoyl carnitine	7.141	304.2383	1.15	1.92×10^{-23}	1.54×10^{-22}	−1.8644	1.0335
PG(38:7)	6.4325	408.7538	18.20	7.18×10^{-24}	6.25×10^{-23}	2.6048	1.1007
PS(46:1)	5.7625	477.3462	−0.77	1.19×10^{-25}	1.97×10^{-24}	−5.9980	1.8726
DAG(37:6)	5.917	645.497	−27.10	8.25×10^{-27}	3.13×10^{-25}	−5.9339	1.8622
DAG(44:11)	5.8551	733.5469	7.15	2.29×10^{-27}	1.69×10^{-25}	−7.1633	1.9705
**Benign vs. Malignant** (borderline +malignant)							
Alanyl-Leucine	0.5998	185.1266	−9.82	0.0299	0.3310	0.4742	1.0870
Malonyl carnitine	3.0338	230.1032	6.51	0.0001	0.1225	−0.4799	2.2927
5-Methoxytryptophan	3.1524	217.098	5.90	0.0141	0.3023	0.5266	2.0353
Tetradecanal	3.2267	231.2506	8.82	0.0072	0.2724	0.1111	1.3363
(Z)-3-Dodecene	3.2438	186.2208	0.81	0.0459	0.3749	0.7191	1.2834
1-(2,4,12-Octadecatrienoyl)piperidine	3.2758	363.329	5.47	0.0214	0.3194	0.6933	1.5567
Cyclocalamin	3.3263	521.2631	12.87	0.0064	0.2724	−0.5958	2.8949
Stearic acid	3.8492	303.3076	4.01	0.0230	0.3272	0.7080	1.4202
Brassica napus non-fluorescent chlorophyll catabolite 3	4.1932	614.2705	4.02	0.0132	0.3023	−0.3264	2.5323
Dihydrolipoamide	4.2231	438.1456	5.81	0.0275	0.3310	−0.2948	1.8247
Arachidic acid	4.4395	332.3389	6.94	0.0155	0.3023	0.4871	1.3481
3-Hydroxyoctanoic acid	4.5635	183.1014	15.66	0.0132	0.3023	−0.7618	2.4363
HMDB0129843	4.6171	688.2496	10.22	0.0066	0.2724	−0.3126	2.3146
N-Acetylaspartylglutamic acid	4.623	327.0775	−6.03	0.0360	0.3583	0.6257	1.2934
Ginsenoyne C	4.9177	277.1789	1.26	0.0375	0.3583	0.5074	1.1694
Behenic acid	4.9745	358.3667	0.59	0.0375	0.3583	0.6200	1.6773
Cerebronic acid	4.9888	402.3927	0.25	0.0326	0.3441	1.0007	2.0765
Thromboxane	5.0003	316.3472	0.95	0.0255	0.3310	0.4727	1.3378
DAG(18:0)	5.2985	395.2757	1.83	0.0126	0.3023	0.6129	1.2636
PE(36:4)	5.5261	722.5014	−8.56	0.0170	0.3023	0.7197	1.4490

Nonoxynol-9	5.5593	635.4548	1.94	0.0162	0.3023	0.5529	1.3716
Tangeraxanthin	5.6027	502.3711	13.44	0.0424	0.3614	0.5039	1.2433
3-(5,6,6-Trimethylbicyclo[2.2.1]hept-1-yl)cyclohexanol	5.6605	254.247	9.38	0.0192	0.3194	0.2245	1.0088
Adrenoyl ethanolamide	5.8872	376.3178	−5.57	0.0062	0.2724	0.4580	1.3109
Arbutin	5.8908	273.0963	1.67	0.0401	0.3583	0.7053	1.4460
Acetyl tributyl citrate	5.903	425.2143	−0.56	0.0153	0.3023	0.6616	1.4220
1-docosene	5.9144	329.3851	−0.04	0.0418	0.3611	0.5375	1.6100
13'-Carboxy-gamma-tocopherol	6.0073	429.317	−22.08	0.0385	0.3583	0.5304	1.2075
Polyethylene, oxidized	6.3653	227.1279	5.17	0.0137	0.3023	−0.5080	2.1763
Azaspiracid 4	6.4618	433.7405	5.58	0.0271	0.3310	0.6277	1.4438
Palmitic amide	6.491	256.2634	8.40	0.0211	0.3194	0.3673	1.4720
Ecgonine methyl ester	6.5117	200.1279	0.46	0.0385	0.3583	−0.3748	1.8902
(±)-(Z)-2-(5-Tetradecenyl)cyclobutanone	6.816	282.2789	7.55	0.0380	0.3583	0.1746	1.2628
18-Hydroxycortisol	6.8204	396.2372	−0.73	0.0097	0.2918	−0.5221	1.8771
MG(18:3)	6.9487	353.2662	−5.09	0.0478	0.3809	0.5997	1.2193
Capryloylglycine	6.9494	202.1437	−1.03	0.0122	0.3023	−0.4321	1.8646
2-trans,4-cis-Decadienoyl carnitine	7.0777	312.2162	−0.15	0.0139	0.3023	−0.2665	1.6030
(ent-16alpha)-16-Kauranol	7.0928	308.2937	0.06	0.0227	0.3272	0.5592	1.2880
Pubescenol	7.1438	475.2956	−10.33	0.0271	0.3310	0.5709	1.3206
Cerulenin	7.2691	224.1282	6.21	0.0207	0.3194	−0.6254	2.3408
3-Oxocholic acid	7.281	407.2759	4.85	0.0092	0.2918	0.6372	1.4820
Ursolic acid	7.3843	495.3321	14.06	0.0009	0.1470	0.6529	2.0814
Homodihydrojasmone	7.553	181.1579	0.02	0.0175	0.3030	1.0793	1.9810
PIP2(32:0)	7.5747	971.4684	4.20	0.0019	0.1916	−0.7850	3.5149
Androsterone glucuronide	7.5776	485.2916	−0.04	0.0018	0.1916	−0.4911	2.4690
5a-Androst-3-en-17-one	7.5895	273.2212	3.23	0.0004	0.1225	−0.7306	3.1692
Pregnanediol-3-glucuronide	7.7812	514.3371	2.81	0.0413	0.3611	−1.1622	2.4896
Geranylcitronellol	7.9792	310.3101	5.97	0.0067	0.2724	0.4903	1.6144
MG(18:0)	8.2383	381.2975	6.90	0.0360	0.3583	0.6689	1.4341
Farnesol	9.1546	240.2321	0.93	0.0283	0.3310	0.6425	1.3005
Geranylacetone	9.1587	212.2008	6.52	0.0331	0.3462	0.5925	1.0961
**Borderline vs. Malignant**							
(E)-Casimiroedine	6.0093	436.2202	−1.56	0.0008	0.6276	−0.5977	2.1053
Jasmonic acid	6.242	211.1319	6.06	0.0034	0.6276	−0.7781	2.6184
9-cis-Retinoic acid	7.3522	323.1956	−7.09	0.0085	0.6276	−0.4697	1.6711
Gibberellin A32	0.9482	397.1659	−6.39	0.0095	0.6276	−0.5518	1.6953
Coniferyl alcohol	5.2862	181.0855	4.04	0.0123	0.6276	−0.7280	2.2225
16a-Hydroxyestrone	5.0385	269.1495	−13.21	0.0127	0.6276	−0.7163	2.1758
Cerulenin	7.2691	224.1282	6.21	0.0132	0.6276	−0.5413	1.6339
Tricyclazole	2.6366	190.0499	−18.12	0.0236	0.6276	−0.4948	1.9763
Indoleacrylic acid	5.4391	171.0637	3.20	0.0286	0.6276	−0.4444	1.5046
Ganglioside GM3 (d18:1/16:0)	6.7729	577.8698	4.06	0.0305	0.6276	0.9515	2.6159
Myrigalone E	7.301	343.1771	8.18	0.0414	0.6276	1.3192	1.1950
Hebevinoside V	8.389	873.5137	10.86	0.0426	0.6276	−0.5576	1.7093

Statistical analysis of differential urine metabolites from the comparison of normal control versus ovarian tumor, benign versus malignant, and borderline versus malignant. RT: Retention Time; *m/z*: Mass-to-Charge Ratio; Delta: the mass accuracy to the theoretical m/z and the detected ion; log2FC: log2 Fold Change.

Table S3. Statistical analysis of differential plasma metabolites from three group of comparison.

Metabolites	RT (min)	*m*/*z*	Delta (ppm)	*P*-value	FDR	log2FC	VIP
**Normal vs. Ovarian tumor**							
Histidinyl-Glycine	0.7099	195.0885	4.35	2.08×10^{-08}	7.91×10^{-07}	−1.4693	2.5525
PA(33:1)	5.5356	684.44	−17.27	3.27×10^{-14}	2.13×10^{-11}	−0.9565	2.5937
PE(20:3/16:1)	5.5344	723.5061	−11.14	4.79×10^{-13}	1.56×10^{-10}	−1.7480	3.3841
Tangeraxanthin	5.5988	507.3342	11.93	1.03×10^{-10}	1.04×10^{-08}	−0.8504	2.2365
Palmitoleoyl Ethanolamide	5.0875	320.257	23.60	1.41×10^{-10}	1.31×10^{-08}	2.0779	3.7986
Crassostrea Secocarotenoid	5.5343	634.4538	10.49	1.54×10^{-10}	1.34×10^{-08}	−0.8483	2.1377
3'-N-Acetyl-4'-O-(14-methylpentadecanoyl)fusarochromanone	5.5666	590.4271	16.70	1.69×10^{-10}	1.38×10^{-08}	−0.7007	1.9424
Palmitic acid	3.2026	275.2771	−0.81	5.28×10^{-10}	3.43×10^{-08}	−0.8149	1.8854
Tetradec-2-enal	5.3095	228.2328	1.06	1.26×10^{-09}	7.11×10^{-08}	3.3895	4.7025
PE(36:4)	5.5344	722.5017	−9.23	1.81×10^{-09}	9.34×10^{-08}	−1.7952	2.9346
Pentadecanol	6.8699	270.275	1.46	1.81×10^{-09}	9.34×10^{-08}	2.9234	4.0249
5,10-Pentadecadien-1-ol	5.7435	242.2488	2.14	9.80×10^{-09}	4.40×10^{-07}	3.2795	4.5598
PE(36:2)	5.5022	766.5302	−4.69	2.19×10^{-08}	7.91×10^{-07}	−2.1049	3.3225
PA(30:2)	5.5666	639.4106	13.29	2.25×10^{-08}	7.91×10^{-07}	−0.6187	1.8122
3-hydroxydecanoyl carnitine	6.9701	332.2427	0.04	2.03×10^{-08}	7.91×10^{-07}	−0.8777	2.3170
PE(34:1)	9.4424	725.0521	7.30	4.12×10^{-08}	1.41×10^{-06}	3.8928	3.4679
Nonoxynol-9	5.5362	617.4242	−0.43	7.26×10^{-08}	2.10×10^{-06}	−0.5282	1.7829
Asiaticoside B	5.5062	508.233	−14.16	2.12×10^{-07}	5.31×10^{-06}	0.8169	3.0373
Oleamide	6.7295	304.2608	10.07	3.21×10^{-07}	7.46×10^{-06}	1.4108	2.7749
Methoxamine	0.8522	229.1543	0.77	3.52×10^{-07}	7.89×10^{-06}	0.5152	1.4005
Palmitic amide	6.4351	256.263	8.45	4.83×10^{-07}	1.03×10^{-05}	1.6095	2.8217
Sphinganine	7.7069	285.2913	0.55	7.36×10^{-07}	1.43×10^{-05}	1.0774	2.5351
L-Octanoyl carnitine	6.6159	288.2161	1.64	8.58×10^{-07}	1.60×10^{-05}	−0.6758	2.1990
(R)-Carvotanacetone	6.7406	170.1543	0.63	1.24×10^{-06}	2.24×10^{-05}	1.3702	2.4099
Methylisopelletierine	6.7406	156.1383	−0.35	1.98×10^{-06}	3.44×10^{-05}	1.0802	2.0901
Tanacetol B	7.3407	314.2328	0.12	2.02×10^{-06}	3.46×10^{-05}	−0.6552	1.8114
Hericene B	5.9364	600.4671	11.00	4.70×10^{-06}	7.28×10^{-05}	−0.0784	1.3611
3-Methyl-2-cyclohexen-1-one	6.7399	128.1071	−0.15	5.30×10^{-06}	7.93×10^{-05}	0.8093	1.7352
Glycocholic acid	7.5224	252.6351	−18.74	7.28×10^{-06}	0.0001	0.3278	2.1794
L-Phenylalanine	6.7759	166.0867	1.53	2.16×10^{-05}	0.0003	0.5274	1.5813
Uridine triphosphate	0.8545	501.9937	2.49	3.18×10^{-05}	0.0004	1.4136	1.9944
Decyl alcohol	4.3663	200.2028	0.82	3.67×10^{-05}	0.0005	1.0245	1.6975
Vitamin A2 aldehyde	4.2155	285.2079	−15.17	4.09×10^{-05}	0.0005	0.3255	1.1528
2-(5,8-Tetradecadienyl)cyclobutanone	6.9658	263.2377	24.45	6.13×10^{-05}	0.0007	−0.4655	1.5205
3-hydroxydodecanoyl carnitine	7.9206	342.2634	−0.22	7.54×10^{-05}	0.0009	−0.4789	1.6009
6,10,14-Trimethyl-5,9,13-pentadecatrien-2-one	6.976	245.2256	0.36	7.68×10^{-05}	0.0009	−0.4335	1.3912
Austalide L	4.8102	447.0557	13.77	0.0001	0.0011	−0.5927	1.5482
8,11,14-Eicosatrienoic acid	7.3424	307.264	−1.50	0.0002	0.0016	−0.4181	1.2458
6-Hydroxyoctadecanoic acid	3.2348	318.2997	−1.10	0.0002	0.0019	−0.3866	1.1473
Dodecanoic acid	6.3903	218.2114	7.55	0.0002	0.0022	−0.4288	1.3916
3-(L-Menthoxy)-2-methylpropane-1,2-diol	6.5658	262.2373	−1.42	0.0003	0.0026	0.2195	1.1396
N-Acetyl aminooctanoic acid	5.5042	184.1327	−0.26	0.0003	0.0027	0.5370	2.3786
5-O-phosphonato-alpha-D-ribofuranosyl Diphosphate(5-)	0.802	372.9602	26.49	0.0004	0.0033	0.9782	2.2707
Enterodiol	5.8883	347.1248	2.22	0.0004	0.0035	−0.7270	2.7618

2-trans,4-cis-Decadienoyl carnitine	7.0519	312.2159	0.45	0.0004	0.0036	–0.4324	1.2384
Phaseolic acid	0.7259	245.1358	–8.60	0.0004	0.0037	0.4169	1.1192
2-Hydroxybenzaldehyde	2.9482	105.0336	–0.64	0.0005	0.0039	–1.0698	1.8632
6-Hydroxyhexadecanoic acid	3.2348	290.2677	21.02	0.0009	0.0069	–1.2459	1.4892
(±)-(Z)-2-(5-Tetradecenyl)cyclobutanone	7.8162	265.2535	0.49	0.0011	0.0084	–0.3909	1.5146
Elaidic acid	6.7435	283.2656	1.08	0.0023	0.0153	1.3132	1.7879
L-Carnitine	0.7272	162.1121	–0.38	0.0026	0.0169	0.3008	1.0947
Phenylacetyl glutamine	4.9737	247.108	3.51	0.0029	0.0187	0.3016	1.0205
alpha-Terpineol formate	0.8064	200.1619	1.68	0.0035	0.0220	–6.4035	3.9481
Tetrahydroharmol	3.1067	204.124	17.37	0.0040	0.0244	–6.4909	4.3331
Neodiospyrin	2.9439	397.071	6.53	0.0064	0.0357	–1.2587	1.8134
alpha-Terpineol propanoate	2.9775	229.199	7.56	0.0195	0.0886	–0.4971	1.1261
**Benign vs. Malignant** (borderline+malignant)							
5-O-phosphonato-alpha-D-ribofuranosyl Diphosphate(5-)	372.9602	372.9602	26.49	0.0227	0.3283	–0.3985	3.0308
Uridine triphosphate	501.9937	501.9937	2.49	0.0340	0.3486	–0.4253	3.1175
5'-O-Methylmelledonal	485.158	485.158	3.19	0.0248	0.3291	–0.3308	1.4676
Tryptophyl-Tyrosine	409.1868	409.1868	0.15	0.0146	0.2873	–0.2541	2.0151
L-Tryptophan	206.1005	206.1005	0.01	0.0244	0.3291	–0.2160	1.9539
3-Oxodecanoic acid	209.1176	209.1176	12.75	0.0021	0.1812	–0.4134	2.0723
3,4-Dihydroxymandelic acid	185.0443	185.0443	0.29	0.0360	0.3513	–0.5068	1.5459
2-Arachidonylglycerol	396.3084	396.3084	1.04	0.0317	0.3458	0.6066	1.1086
Lucidenic acid A	476.306	476.306	12.25	0.0139	0.2868	1.1363	1.4422
3,7-Dihydroxy-25-methoxycucurbita-5,23-dien-19-al	528.406	528.406	10.75	0.0007	0.1812	0.3942	1.1321
PA(30:2)	639.4106	639.4106	13.29	0.0079	0.2623	0.4084	1.2376
Okadaic acid	828.4469	828.4469	–16.28	0.0010	0.1812	0.6162	2.5672
1,7-Dimethyl guanosine	330.1642	330.1642	10.90	0.0041	0.2189	0.4236	1.8014
Arbutin	273.0984	273.0984	1.66	0.0160	0.2982	0.3643	1.5403
Estrone	271.1652	271.1652	–14.00	0.0407	0.3604	0.4901	1.1105
2-trans,4-cis-Decadienoyl carnitine	312.2159	312.2159	0.45	0.0126	0.2780	0.4928	1.1053
Tanacetol B	314.2328	314.2328	0.12	0.0195	0.3037	0.6986	1.2132
Alpha-Linolenic acid	292.2609	292.2609	1.77	0.0035	0.2178	0.1680	1.4742
3-hydroxydodecanoyl carnitine	342.2634	342.2634	–0.22	0.0287	0.3458	0.7715	1.0036
**Borderline vs. Malignant**							
Rutacridone	2.3507	637.2364	5.31	0.0002	0.1191	–0.5052	2.2425
Methoxamine	0.8522	229.1543	0.77	0.0067	0.2195	0.2031	1.5321
8,11,14-Eicosatrienoic acid	7.3424	307.264	–1.50	0.0082	0.2419	–0.5519	2.0917
Phenylacetyl glutamine	4.9737	247.108	3.51	0.0132	0.2912	–0.4181	1.4345
Sphinganine	7.7069	285.2913	0.55	0.0207	0.3636	0.6217	2.2933
5'-O-Methylmelledonal	1.5925	485.158	3.19	0.0378	0.4925	–0.5424	1.5001
Elaidic acid	6.7435	283.2656	1.08	0.0378	0.4925	1.3700	3.6240
5-O-phosphonato-alpha-D-ribofuranosyl Diphosphate(5-)	0.802	372.9602	26.49	0.0390	0.4977	0.8051	2.9858
Uridine triphosphate	0.8545	501.9937	2.49	0.0480	0.5478	0.6359	2.2719
2-Arachidonylglycerol	3.9906	396.3084	1.04	0.0494	0.5494	–0.9633	1.5881

Statistical analysis of differential plasma metabolites from the comparison of normal control versus ovarian tumor, benign versus malignant, and borderline versus malignant. RT: Retention Time; *m/z*: Mass-to-Charge Ratio; Delta: the mass accuracy to the theoretical m/z and the detected ion; log2FC: log2 Fold Change.

© 2020 by the authors. Licensee MDPI, Basel, Switzerland. This article is an open access article distributed under the terms and conditions of the Creative Commons Attribution (CC BY) license (http://creativecommons.org/licenses/by/4.0/).
